# Epidemiology of pre-existing multimorbidity in pregnant women in the UK in 2018: a population-based cross-sectional study

**DOI:** 10.1186/s12884-022-04442-3

**Published:** 2022-02-11

**Authors:** Siang Ing Lee, Amaya Azcoaga-Lorenzo, Utkarsh Agrawal, Jonathan I. Kennedy, Adeniyi Francis Fagbamigbe, Holly Hope, Anuradhaa Subramanian, Astha Anand, Beck Taylor, Catherine Nelson-Piercy, Christine Damase-Michel, Christopher Yau, Francesca Crowe, Gillian Santorelli, Kelly-Ann Eastwood, Zoe Vowles, Maria Loane, Ngawai Moss, Peter Brocklehurst, Rachel Plachcinski, Shakila Thangaratinam, Mairead Black, Dermot O’Reilly, Kathryn M. Abel, Sinead Brophy, Krishnarajah Nirantharakumar, Colin McCowan, on behalf of the MuM-PreDiCT Group

**Affiliations:** 1 Institute of Applied Health Research, IOEM Building, University of Birmingham, Edgbaston, Birmingham, B15 2TT UK; 2 Division of Population and Behavioural Sciences, School of Medicine, University of St Andrews, St Andrews, UK; 3 Data Science, Medical School, Swansea University, Swansea, UK; 4 Department of Epidemiology and Medical Statistics, College of Medicine, University of Ibadan, Ibadan, Nigeria; 5 Centre for Women’s Mental Health, Division of Psychology and Mental Health, School of Health Sciences, Faculty of Biology Medicine & Health, The University of Manchester, Manchester, UK; 6 Guy’s and St. Thomas’ NHS Foundation Trust, London, UK; 7 Medical and Clinical Pharmacology, School of Medicine, Université Toulouse III, Toulouse, France; 8 INSERM, Centre for Epidemiology and Research in Population Health (CERPOP), CIC 1436 Toulouse, France; 9 Division of Informatics, Imaging and Data Sciences, Faculty of Biology Medicine and Health, The University of Manchester, Manchester, UK; 10 Health Data Research, London, UK; 11 Bradford Institute for Health Research, Bradford, UK; 12 Centre for Public Health, Queen’s University of Belfast, Belfast, UK; 13 St Michael’s Hospital, University Hospitals Bristol NHS Foundation Trust, Bristol, UK; 14 The Institute of Nursing and Health Research, Ulster University, Newtownabbey, UK; 15 Patient and Public Representative, London, UK; 16 WHO Collaborating Centre for Global Women’s Health, Institute of Metabolism and Systems Research, University of Birmingham, Birmingham, UK; 17 Department of Obstetrics and Gynaecology, Birmingham Women’s and Children’s NHS Foundation Trust, Birmingham, UK; 18 Aberdeen Centre for Women’s Health Research, School of Medicine, Medical Science and Nutrition, University of Aberdeen, Aberdeen, UK; 19 Greater Manchester Mental Health NHS Foundation Trust, Manchester, UK

**Keywords:** Multimorbidity, Multiple chronic conditions, Multiple long-term conditions, Pregnancy, Maternity, Epidemiology

## Abstract

**Background:**

Although maternal death is rare in the United Kingdom, 90% of these women had multiple health/social problems. This study aims to estimate the prevalence of pre-existing multimorbidity (two or more long-term physical or mental health conditions) in pregnant women in the United Kingdom (England, Northern Ireland, Wales and Scotland).

**Study design:**

Pregnant women aged 15–49 years with a conception date 1/1/2018 to 31/12/2018 were included in this population-based cross-sectional study, using routine healthcare datasets from primary care: Clinical Practice Research Datalink (CPRD, United Kingdom, *n* = 37,641) and Secure Anonymized Information Linkage databank (SAIL, Wales, *n* = 27,782), and secondary care: Scottish Morbidity Records with linked community prescribing data (SMR, Tayside and Fife, *n* = 6099). Pre-existing multimorbidity preconception was defined from 79 long-term health conditions prioritised through a workshop with patient representatives and clinicians.

**Results:**

The prevalence of multimorbidity was 44.2% (95% CI 43.7–44.7%), 46.2% (45.6–46.8%) and 19.8% (18.8–20.8%) in CPRD, SAIL and SMR respectively. When limited to health conditions that were active in the year before pregnancy, the prevalence of multimorbidity was still high (24.2% [23.8–24.6%], 23.5% [23.0–24.0%] and 17.0% [16.0 to 17.9%] in the respective datasets). Mental health conditions were highly prevalent and involved 70% of multimorbidity CPRD: multimorbidity with ≥one mental health condition/s 31.3% [30.8–31.8%]).

After adjusting for age, ethnicity, gravidity, index of multiple deprivation, body mass index and smoking, logistic regression showed that pregnant women with multimorbidity were more likely to be older (CPRD England, adjusted OR 1.81 [95% CI 1.04–3.17] 45–49 years vs 15–19 years), multigravid (1.68 [1.50–1.89] gravidity ≥ five vs one), have raised body mass index (1.59 [1.44–1.76], body mass index 30+ vs body mass index 18.5–24.9) and smoked preconception (1.61 [1.46–1.77) vs non-smoker).

**Conclusion:**

Multimorbidity is prevalent in pregnant women in the United Kingdom, they are more likely to be older, multigravid, have raised body mass index and smoked preconception. Secondary care and community prescribing dataset may only capture the severe spectrum of health conditions. Research is needed urgently to quantify the consequences of maternal multimorbidity for both mothers and children.

**Supplementary Information:**

The online version contains supplementary material available at 10.1186/s12884-022-04442-3.

## Background

Multimorbidity is having two or more long-term health conditions [1]. Although well studied in other disease area, there is currently sparse literature for multimorbidity in pregnant women. Pregnant women with multimorbidity are at increased risk of adverse outcomes for mother and child [2, 3]. Although maternal death is rare in the United Kingdom (UK), 90 % of women who died during/within a year after pregnancy had multiple health and social problems [4].

Multimorbidity increases health care burden for patients, for instance, needing to attend multiple health care appointments and being on multiple medications [1]. These challenges increase during pregnancy, with the addition of specialist antenatal clinic appointments and monitoring, and concerns regarding how medications may affect the developing foetus.

Despite this, there is a dearth of basic information on the prevalence and types of pre-existing health conditions affecting pregnant women. Better understanding of the epidemiology of multimorbidity in pregnant women could help policy makers and health care providers plan services to prevent women from developing multimorbidity, for early detection and optimal management of health conditions prior to conception, and tailor maternity services to pregnant women with multimorbidity.

In the UK, most people are registered with a general practitioner (GP), the gatekeepers to primary care and specialist referrals. In secondary care, health care utilization administrative data are recorded for reimbursement. Thus, both provide good data sources for multimorbidity and pregnancy research.

This study aims to describe the epidemiology of pre-existing multimorbidity in pregnant women. It also seeks to understand the utility of routine health care datasets in the study of multimorbidity in pregnant women, by using three datasets from different health care settings and across the four UK nations (England, Northern Ireland, Scotland and Wales).

## Methods

### Study design and study period

This was a cross sectional analysis of the prevalence of pre-existing multimorbidity prior to the start of pregnancy in the UK across three separate databases. We included index pregnancies where the conception date was between 1/1/2018 and 31/12/2018.

### Inclusion and exclusion criteria

Women aged 15–49 years with a conception date in 2018 were eligible. Last menstrual period or gestational day 0 was considered the conception date [5]. When a woman had more than one pregnancy episode in 2018, the first recorded pregnancy in that year was included (not necessarily the first ever pregnancy). Women whose data did not meet standard quality checks were excluded (Additional file 1).

### Data sources

This study used three datasets from different health settings, covering all four nations in the UK: Clinical Practice Research Datalink, (CPRD, England, Northern Ireland, Scotland and Wales), Secure Anonymized Information Linkage (SAIL, Wales) and Scottish Morbidity Records (SMR, Scotland).

#### Primary care

CPRD GOLD contains anonymized, longitudinal medical records for over 19 million patients in the UK (England, Northern Ireland, Scotland and Wales) from over 940 participating general practices; it currently covers 4% of UK GP practices and is widely acknowledged to be representative of the UK population [6]. It includes data on demographics, diagnoses and prescriptions [6]. Linkage to area based deprivation index was available for patients in England. Within CPRD GOLD, the CPRD Pregnancy Register is an algorithm that takes information from maternity, antenatal and delivery health records to detect pregnancy episodes and their outcomes [5].

The SAIL databank is a whole population level database in Wales. It is a repository of anonymized health and socio-economic administrative data and provides linkage at an individual level [7]. It holds data for 4.8 million people and covers 80% of Welsh GP practices [7]. Within SAIL, the National Community Child Health Dataset was used to detect pregnancies and was linked to the Welsh Longitudinal General Practice dataset and the Welsh Demographic Service dataset for diagnoses, prescriptions and demographics data respectively.

#### Secondary care and community prescriptions

SMR data was available from two Scottish regional health boards: National Health Service (NHS) Tayside and NHS Fife [8]. A dataset was created linking the Scottish Maternity Records (SMR02) to data from Hospital Admissions (SMR01), Mental Health Inpatients (SMR04), Accident and Emergency, and the Demography and Death registry. This covered diagnoses and demographic data for all inpatient stays and day cases for residents in the two regions. The dataset was also linked to the Prescribing Information System for data on all medications dispensed in the community. Pregnancies were detected from maternity records or pregnancy-related hospital admissions.

### Definition of multimorbidity

Multimorbidity was defined by the presence of two or more pre-existing long-term physical or mental health conditions prior to the index pregnancy. We defined long-term conditions as conditions that have ongoing significant impact on patients, including conditions that are relapsing and remitting in nature.

One of the wider research aims is to mitigate the effect of multimorbidity on adverse pregnancy outcomes. As pregnancy related conditions (e.g., gestational diabetes and pregnancy induced hypertension) will be subsequently studied as maternal outcomes, they were not included in the definition of pre-existing multimorbidity.

An exhaustive list of long-term health conditions was first identified from existing literature [4, 9, 10], in particular based on the work commissioned by Health Data Research UK on multimorbidity conceptualization [10] and health conditions that were leading indirect cause of death in the UK maternal mortality report (MBRRACE) [4]. This list and phenome definitions were refined and harmonized through workshops with our research advisory group, consisting of patient and public representatives, clinicians from general practice, obstetrics, maternal medicine, psychiatry, public health, and data scientists. Seventy nine health conditions were selected on the following basis: (i) prevalence; (ii) potential to impact on pregnancy outcomes; (iii) considered important by women; and (iv) recorded in the study datasets.

Diagnoses of these 79 long-term health conditions were determined from Read Codes version 2 (primary care datasets) and the International Classification of Disease 10th version (secondary care datasets) [11]. The validity of diagnostic coding has previously been shown to be good in primary care records and generally health conditions under payment for performance schemes, such as Quality Outcomes Framework, are well coded [12]. Code lists and phenome definitions used are available in Additional files 2 and 3.

### Data analysis

The primary analysis was the prevalence of pre-existing multimorbidity in pregnant women. The denominator was the total number of index pregnancies identified in 2018, regardless of the pregnancy outcome. Additional analysis was performed for multimorbidity with at least one mental health conditions and active multimorbidity. Active multimorbidity limits common transient/episodic conditions (e.g., mental health, dermatological and atopic conditions and headaches) to those that were active in the 12 months preceding index pregnancy (Additional file 3).

Multivariable logistic regression was performed to examine the association of multimorbidity with maternal age (five-yearly categories), ethnicity, deprivation quintiles (patient level Index of Multiple Deprivation [IMD] for all three datasets), latest maternal pre-pregnancy body mass index (BMI) categories, latest pre-pregnancy smoking status, and gravidity (total number of pregnancies up to and including index pregnancy). Obesity was considered a covariate (BMI categories) instead of a health condition. For CPRD, practice level IMD was available for all four nations, but patient level IMD was only available for England, therefore, the regression analysis was limited to England. We then described the prevalence of individual health conditions, and the prevalence of mutually exclusive multimorbidity combinations.

Missing data were assigned to separate categories and included in the analyses. Sensitivity analysis was performed for CPRD (England), where missing ethnicity was imputed with data from linked hospital administrative data, and missing patient level IMD was imputed with practice level IMD.

Study results were presented for each dataset separately. Data were not combined as there was a possibility of patient overlap between CPRD (Wales, Scotland) with both SAIL (Wales) and SMR (Scotland). Deduplication was not possible as the datasets are anonymized, and only aggregated data were exported within the permission of the data access approval.

### Post hoc analysis

As our study found no association of recorded multimorbidity with social deprivation, we conducted a post hoc analysis in the CPRD cohort, with the list of conditions used to define multimorbidity in a seminal paper that found this association [13]. We also examined the association of selected health conditions with deprivation and ethnicity. Guided by our patient representatives, we analysed the prevalence of multimorbidity for selected health conditions to illustrate the burden of multimorbidity. The selected health conditions were: i) the top ten most common individual health conditions in this study, and ii) leading causes of maternal deaths [4].

Analysis was performed using STATA 16 and R. The study is reported in accordance with the RECORD guideline (Additional file 4).

## Results

### Study population

Overall, there were 37,641 (CPRD), 27,782 (SAIL) and 6099 (SMR) pregnant women aged 15–49 years included in the analysis in 2018. Additional Fig. 1 presents the flow chart for the study population selection. The characteristics of the study cohort is presented in Table 1 and Additional Table 1. Most of the study participants were 20–34 years old, of White ethnicity, of normal weight or were overweight pre-pregnancy and were non-smoker pre-pregnancy. Linkage to area-based data for patient level IMD was available for 75% of the CPRD study cohort in England. There were more affluent women in the primary care dataset but vice versa for SMR.

**Table 1:** Baseline characteristics of pregnant women in CPRD (UK), SAIL (Wales) and SMR (Scotland) in 2018

Characteristics	Frequency (percentage)					
	CPRD (UK)		SAIL (Wales)		SMR (Scotland)	
**Total**	37,641	–	27,782	–	6099	–
**Nation**						
England	13,075	(34.74%)	–	–	–	–
Northern Ireland	2984	(7.93%)	–	–	–	–
Scotland	12,559	(33.37%)	–	–	–	–
Wales	9023	(23.97%)	–	–	–	–
**Age categories (5 yearly)**						
15–19	2534	(6.73%)	1537	(5.53%)	422	(6.92%)
20–24	6604	(17.54%)	5360	(19.29%)	1147	(18.81%)
25–29	10,204	(27.11%)	8617	(31.02%)	1830	(30.00%)
30–34	10,723	(28.49%)	8081	(29.09%)	1746	(28.63%)
35–39	5970	(15.86%)	3549	(12.77%)	803	(13.17%)
40–44	1428	(3.79%)	603	(2.17%)	138	(2.26%)
45–49	178	(0.47%)	35	(0.13%)	13	(0.21%)
**Gravidity**						
1	11,480	(30.50%)	13,006	(46.81%)	1800	(29.51%)
2	9895	(26.29%)	9972	(35.89%)	1992	(32.66%)
3	6734	(17.89%)	3252	(11.71%)	1105	(18.12%)
4	4004	(10.64%)	1035	(3.73%)	580	(9.51%)
≥ 5	5528	(14.69%)	517	(1.86%)	618	(10.13%)
Missing	–	–	–	–	4	(0.07%)
**Ethnicity**						
Asian / South Asians^a^	1261	(3.35%)	418	(1.50%)	149	(2.44%)
Black	973	(2.58%)	178	(0.64%)	23	(0.38%)
Mixed	305	(0.81%)	121	(0.44%)	8	(0.13%)
Other	528	(1.40%)	229	(0.82%)	91	(1.49%)
White	20,818	(55.31%)	17,430	(62.74%)	4903	(80.39%)
Missing	13,756	(36.55%)	9406	(33.86%)	925	(15.17%)
**BMI (kg/m**^**2**^**)**						
Underweight (< 18.5)	1217	(3.23%)	1287	(4.63%)	92	(1.51%)
Normal Weight (18.5–24.9)	14,440	(38.36%)	9485	(34.14%)	1478	(24.23%)
Overweight (25–29.9)	8075	(21.45%)	5658	(20.37%)	1010	(16.56%)
Obese (30+)	7178	(19.07%)	5372	(19.34%)	1279	(20.97%)
Missing	6731	(17.88%)	5980	(21.52%)	2240	(36.73%)
**Smoking**						
Non-Smoker	22,395	(59.50%)	10,151	(36.54%)	3349	(54.91%)
Ex-smoker	5707	(15.16%)	8022	(28.87%)	863	(14.15%)
Smoker	8237	(21.88%)	6612	(23.80%)	1041	(17.07%)
Missing	1302	(3.46%)	2997	(10.79%)	846	(13.87%)
**Patient level deprivation quintiles (IMD)**	**Only available for England**^**b**^					
1, least deprived	2326	(17.79%)	6455	(23.23%)	722	(11.84%)
2	1835	(14.03%)	5460	(19.65%)	1039	(17.04%)
3	1878	(14.36%)	4779	(17.20%)	979	(16.05%)
4	1853	(14.17%)	4032	(14.51%)	1253	(20.54%)
5, most deprived	1908	(14.59%)	3832	(13.79%)	1344	(22.04%)
Missing	3275	(25.05%)	3224	(11.60%)	762	(12.49%)

^a^ South Asian for CPRD, Asian for SAIL and SMR

^b^ Aggregate IMD quintiles cannot be provided for UK as each nation has its specific IMD; data presented here is patient level IMD for England only (*n* = 13,075). Practice level IMD for all four UK nations in CPRD is available in Additional Table 1

*BMI* body mass index, *CPRD* Clinical Practice Research Datalink, *IMD* Index of Multiple Deprivation, *SAIL* The Secure Anonymized Information Linkage databank, *SMR* Scottish Morbidity Records

### Multimorbidity

The prevalence of pre-existing multimorbidity in pregnant women was 44.2% (95% confidence intervals [CI] 43.7 to 44.7%), 46.2% (45.6 to 46.8%) in CPRD and SAIL respectively (primary care dataset) but was halved in SMR’s secondary care and community prescription dataset, 19.8% (18.8 to 20.8%).

Over 70 % of pregnant women with multimorbidity had mental health condition/s: 31.3% (30.8 to 31.8%), 33.7% (33.1 to 34.2%) and 14.6% (16.0 to 17.9%) of pregnant women had multimorbidity with at least one mental health conditions in CPRD, SAIL and SMR respectively. The prevalence of active multimorbidity was half that of the primary analysis in primary care datasets, 24.2% (23.8 to 24.6%) and 23.5% (23.0 to 24.0%) for CPRD and SAIL respectively, but remained similar for SMR, 17.0% (16.0 to 17.9%). The percentage of pregnant women by the total morbidity count is available in Additional Table 2.

### Characteristics associated with multimorbidity

The prevalence of pre-existing multimorbidity by the characteristics of pregnant women is presented in Additional Table 3. In the CPRD England study cohort (*n* = 13,075), when all characteristics were adjusted for, increasing maternal age and gravidity remained significantly associated with multimorbidity (maternal age 45–49 years, adjusted odds ratio [aOR] 1.8, 95% CI 1.0 to 3.2; gravidity ≥5, 1.7, 1.5 to 1.9); pregnant women with BMI 25 to 29.9 (aOR 1.2, 95% CI 1.1 to 1.3), BMI 30+ (1.6, 1.4 to 1.8), were smokers (1.6, 1.5 to 1.8) or ex-smokers (1.4, 1.3 to 1.6) had higher odds of multimorbidity. However, higher odds of multimorbidity were not observed in pregnant women of ethnic minority groups or from more deprived socioeconomic groups (Table 2). Findings were similar in the sensitivity analysis of CPRD (England) using imputed data for missing ethnicity and IMD (Additional Table 4). In SAIL, the effect sizes of characteristics were generally similar to that in CPRD (England).

**Table 2:** Logistic regression for pre-existing multimorbidity by women’s characteristics

Characteristics	CPRD (England), ***n*** = 13,075						SAIL (Wales), ***n*** = 27,782						SMR (Scotland), ***n*** = 6099					
	Unadjusted OR (95% CI)			Adjusted OR (95% CI)			Unadjusted OR (95% CI)			Adjusted OR (95% CI)			Unadjusted OR (95% CI)			Adjusted OR (95% CI)		
**Age categories (5 yearly)**																		
15–19	Ref	–	–	Ref	–	–	Ref	–	–	Ref	–	–	Ref	–	–	Ref	–	–
20–24	1.60	(1.34	−1.90)	1.17	(0.97	− 1.42)	1.64	(1.45	− 1.85)	1.10	(0.96	−1.26)	1.48	(1.11	−1.94)	1.42	(1.05	− 1.92)
25–29	1.80	(1.52	−2.12)	1.21	(1.01	− 1.45)	1.93	(1.72	−2.17)	1.21	(1.06	−1.39)	1.36	(1.02	−1.81)	1.45	(1.07	− 1.95)
30–34	1.84	(1.56	−2.17)	1.26	(1.05	− 1.52)	2.19	(1.94	−2.46)	1.36	(1.19	−1.56)	1.18	(0.89	−1.58)	1.32	(0.97	−1.80)
35–39	1.95	(1.64	−2.32)	1.28	(1.06	−1.56)	2.54	(2.24	− 2.89)	1.64	(1.42	−1.90)	1.23	(0.90	−1.69)	1.37	(0.97	−1.93)
40–44	2.55	(2.04	−3.20)	1.61	(1.26	−2.06)	3.19	(2.63	−3.88)	2.20	(1.77	−2.73)	1.04	(0.62	−1.75)	1.18	(0.68	−2.04)
45–49	2.98	(1.74	−5.11)	1.81	(1.04	−3.17)	3.88	(1.94	−7.76)	4.11	(1.83	−9.26)	1.56	(0.42	−5.87)	1.67	(0.45	−6.93)
**Gravidity**																		
1	Ref	–	–	Ref	–	–	Ref	–	–	Ref	–	–	Ref	–	–	Ref	–	–
2	1.07	(0.97	−1.18)	0.98	(0.89	−1.08)	1.20	(1.14	−1.26)	1.03	(0.97	−1.09)	1.09	(0.92	−1.30)	1.03	(0.86	−1.23)
3	1.35	(1.22	−1.50)	1.18	(1.06	−1.32)	1.51	(1.40	−1.63)	1.16	(1.07	−1.27)	1.30	(1.07	−1.58)	1.17	(0.95	−1.43)
4	1.52	(1.35	−1.72)	1.30	(1.15	−1.48)	1.89	(1.67	−2.15)	1.39	(1.21	−1.60)	1.77	(1.41	−2.21)	1.49	(1.17	−2.01)
≥ 5	2.11	(1.9	−2.35)	1.68	(1.50	−1.89)	2.73	(2.27	−3.29)	1.81	(1.48	−2.22)	2.70	(2.21	−3.34)	2.19	(1.75	−2.76)
Missing	–	–	–	–	–	–	–	–	–	–	–	–	1.75	(0.18	−16.88)	1.31	(0.13	−13.43)
**Ethnicity**																		
Asian /South Asian^a^	0.60	(0.52	−0.69)	0.66	(0.56	−0.77)	0.48	(0.39	−0.59)	0.53	(0.43	−0.66)	0.44	(0.26	−0.75)	0.51	(0.29	−0.87)
Black	0.76	(0.63	−0.91)	0.73	(0.61	− 0.89)	0.31	(0.22	− 0.43)	0.31	(0.22	−0.45)	0.78	(0.27	−2.31)	0.86	(0.29	−2.58)
Mixed	0.88	(0.67	−1.16)	0.95	(0.72	−1.26)	0.78	(0.54	−1.11)	0.86	(0.59	−1.27)	–	–	–	–	–	–
Other	0.55	(0.44	− 0.70)	0.61	(0.48	− 0.77)	0.31	(0.23	− 0.42)	0.33	(0.24	− 0.45)	0.66	(0.38	−1.15)	0.59	(0.33	−1.05)
White	Ref	–	–	Ref	–	–	Ref	–	–	Ref	–	–	Ref	–	–	Ref	–	–
Missing	0.85	(0.78	−0.93)	0.94	(0.86	−1.03)	0.61	(0.58	−0.65)	0.74	(0.70	−0.78)	0.62	(0.51	−0.75)	0.63	(0.52	−0.77)
**BMI (kg/m**^**2**^**)**																		
Underweight (< 18.5)	0.89	(0.73	−1.07)	0.91	(0.75	−1.10)	1.06	(0.94	−1.19)	1.08	(0.96	−1.22)	1.59	(0.96	−2.63)	1.27	(0.68	−1.93)
Normal Weight (18.5–24.9)	Ref	–	–	Ref	–	–	Ref	–	–	Ref	–	–	Ref	–	–	Ref	–	–
Overweight (25–29.9)	1.20	(1.10	−1.31)	1.16	(1.05	−1.27)	1.19	(1.11	−1.27)	1.17	(1.09	−1.25)	1.31	(1.06	−1.62)	1.33	(0.75	−2.16)
Obese (30+)	1.69	(1.53	−1.86)	1.59	(1.44	−1.76)	1.57	(1.47	−1.69)	1.51	(1.41	−1.62)	1.98	(1.64	−2.39)	1.90	(1.55	−2.28)
Missing	0.60	(0.54	−0.67)	0.73	(0.64	−0.82)	0.26	(0.24	−0.28)	0.61	(0.56	−0.67)	1.21	(1.02	−1.44)	1.24	(1.03	−1.49)
**Smoking**																		
Non-Smoker	Ref	–	–	Ref	–	–	Ref	–	–	Ref	–	–	Ref	–	–	Ref	–	–
Ex-Smoker	1.62	(1.47	−1.78)	1.43	(1.29	−1.57)	1.82	(1.71	−1.93)	1.58	(1.49	−1.68)	1.47	(1.22	−1.78)	1.32	(1.10	−1.60)
Smoker	1.69	(1.54	−1.84)	1.61	(1.46	−1.77)	2.19	(2.05	−2.33)	2.03	(1.90	−2.17)	2.54	(2.17	−2.95)	2.06	(1.79	−2.51)
Missing	0.31	(0.24	−0.41)	0.48	(0.36	−0.64)	0.07	(0.06	−0.08)	0.12	(0.10	−0.14)	1.16	(0.95	−1.41)	1.19	(0.96	−1.47)
**Patient level deprivation quintiles (IMD)**																		
1, least deprived	Ref	–	–	Ref	–	–	Ref	–	–	Ref	–	–	Ref	–	–	Ref	–	–
2	0.95	(0.84	−1.07)	0.92	(0.81	−1.05)	0.87	(0.81	−0.93)	0.88	(0.82	−0.96)	1.33	(0.99	−1.77)	1.22	(0.92	−1.64)
3	1.00	(0.88	−1.13)	0.93	(0.82	−1.05)	0.90	(0.83	−0.97)	0.92	(0.85	−1.00)	1.89	(1.40	−2.45)	1.56	(1.17	−2.07)
4	0.98	(0.87	−1.11)	0.90	(0.79	−1.02)	0.78	(0.72	−0.84)	0.87	(0.79	−0.95)	2.67	(2.06	−3.49)	2.18	(1.67	−2.87)
5, most deprived	0.96	(0.85	−1.09)	0.88	(0.77	−1.00)	0.88	(0.82	−0.96)	0.90	(0.83	−0.99)	2.49	(1.89	−3.19)	1.77	(1.34	−2.34)
Missing	0.90	(0.81	−1.00)	0.86	(0.77	−0.96)	0.80	(0.74	−0.88)	0.90	(0.82	−0.99)	1.82	(1.36	−2.44)	1.65	(1.23	−2.23)

^a^ South Asian for CPRD, Asian for SAIL and SMR

Adjusted for maternal age, gravidity, ethnicity, BMI, smoking status, patient level IMD

*BMI* body mass index, *CI* confidence intervals, *CPRD* Clinical Practice Research Datalink, *IMD* Index of Multiple Deprivation, *OR* odds ratio, *Ref* reference group, *SAIL* The Secure Anonymized Information Linkage databank, *SMR* Scottish Morbidity Records

In SMR, after adjusting for all characteristics, higher odds of multimorbidity were observed only in those age 20–24 and 25–29 years, had gravidity of 3+, BMI 30+, were smokers and ex-smokers and those from more deprived socioeconomic groups. The odds of multimorbidity were not higher in ethnic minority groups (Table 2).

### Post hoc analysis

**Fig. 1:**
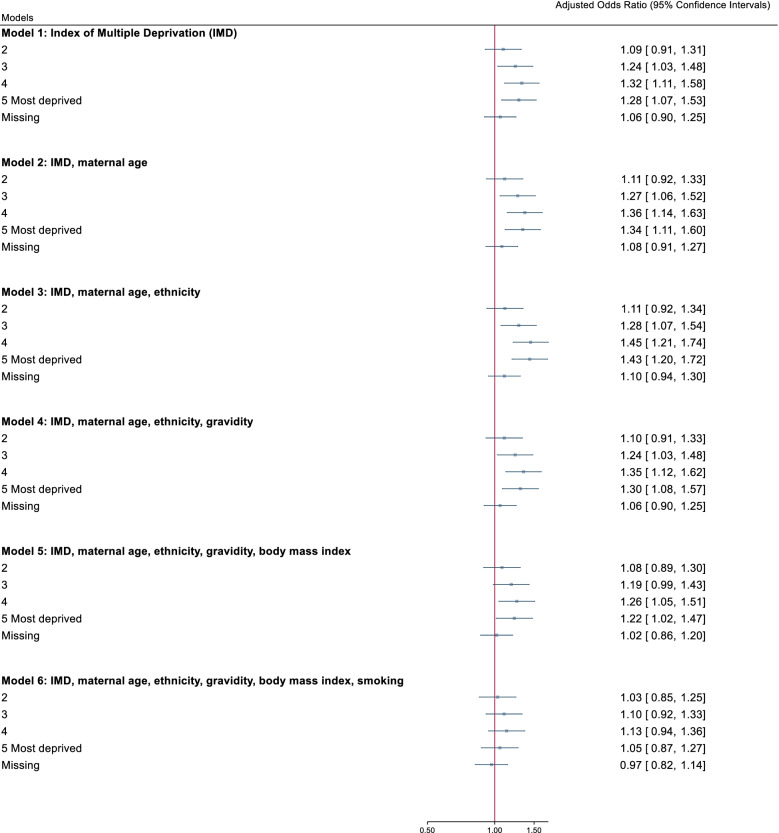
Forest plot of odds ratio for multimorbidity and social deprivation. Legend: Multimorbidity was defined using the 31 health conditions in Barnet et al’s study, logistic regression was used to analyse the study cohort in CPRD England (*n* = 13,075). The reference group was index of multiple deprivation (IMD) quintile 1 (least deprived)

Post hoc analysis was performed to explore whether the lack of association of multimorbidity with deprivation in our primary care datasets was, in part, due to the health conditions we used to define multimorbidity. Logistic regression was repeated in CPRD (England) with the list of 31 health conditions used to define multimorbidity in Barnet et al’s seminal paper [13], the adjusted variables were added in a step-wise manner. After adjusting for maternal age, ethnicity and gravidity, increasing levels of deprivation were associated with higher odds of multimorbidity (most deprived quintile aOR 1.30, 95% CI 1.08 to 1.57). This association was attenuated and was no longer significant when raised BMI and smoking status were added to the model (aOR 1.05, 0.87 to 1.27, Additional Table 5, Fig. 1).

To test this hypothesis further, we repeated the logistic regression in the CPRD (England) cohort by removing eight health conditions that were associated with being in less deprived socioeconomic groups. When adjusted for maternal age, ethnicity and gravidity, multimorbidity (defined by 71 health conditions) was associated with deprivation (most deprived quintile aOR 1.26, 1.10 to 1.44). This association was attenuated and was no longer significant when raised BMI and smoking status were added (aOR 1.08, 0.94 to 1.24, Additional Table 6).

### Individual health conditions

Table 3 presents the top 20 most prevalent health conditions in our study cohort. The top four most common health conditions across all three datasets were depression, anxiety (both known as common mental health disorders), allergic rhinoconjunctivitis and asthma, with the prevalence of common mental health disorders being consistently around 20%. The full list of prevalence for each health condition is presented in Additional Table 7.

**Table 3:** Top 20 most prevalent individual health conditions in pregnant women

CPRD (UK), ***n*** = 37,641					SAIL (Wales), ***n*** = 27,782				SMR (Scotland), ***n*** = 6099			
No	Health conditions	Percentage, % (95% CI)			Health conditions	Percentage, % (95% CI)			Health conditions	Percentage, % (95% CI)		
1	Depression (diagnosis)	23.43	(23.01	−23.87)	Depression (diagnosis)	24.07	(23.57	−24.58)	Common mental health disorders (prescription)	22.66	(21.61	−23.71)
2	Anxiety (diagnosis)	18.98	(18.58	−19.38)	Anxiety (diagnosis)	23.05	(22.56	−23.55)	Asthma	9.94	(9.19	−10.69)
3	Allergic rhinoconjunctivitis	16.35	(15.98	−16.73)	Allergic rhinoconjunctivitis	18.53	(18.08	−19.00)	Allergic rhinoconjunctivitis	7.56	(6.90	−8.22)
4	Asthma	14.63	(14.27	−14.99)	Asthma	17.17	(16.73	−17.62)	Peptic ulcer disease/GORD	6.98	(6.35	−7.62)
5	Migraine	12.71	(12.38	−13.05)	Migraine	13.47	(13.07	−13.88)	Atopic eczema	6.35	(5.73	−6.96)
6	Other mental health conditions	8.85	(8.57	−9.14)	Other mental health conditions	9.43	(9.09	−9.78)	Other mental health conditions	4.84	(4.30	−5.38)
7	IBS	7.97	(7.70	−8.25)	IBS	7.83	(7.52	−8.15)	Migraine	3.69	(3.22	−4.16)
8	Other skin conditions	5.47	(5.25	−5.71)	Other chronic headaches	6.60	(6.31	−6.90)	IBS	3.16	(2.73	−3.60)
9	PCOS	4.66	(4.45	−4.88)	Other skin conditions	5.71	(5.44	−5.98)	Thyroid disorder	3.12	(2.68	−3.55)
10	Psoriasis	3.90	(3.70	−4.10)	Atopic eczema	3.97	(3.74	−4.20)	Severe mental illness	2.26	(1.89	−2.64)
11	Female infertility	3.81	(3.62	−4.01)	PCOS	3.96	(3.73	−4.20)	Cholelithiasis	2.15	(1.78	−2.51)
12	Other chronic headaches	3.53	(3.35	−3.72)	Psoriasis	3.62	(3.41	−3.85)	Depression (diagnosis)	1.71	(1.38	−2.03)
13	Thyroid disorder	3.34	(3.16	−3.52)	Thyroid disorder	2.45	(2.28	−2.64)	Epilepsy	1.57	(1.26	−1.89)
14	Atopic eczema	3.06	(2.89	−3.24)	Alcohol misuse	2.25	(2.08	−2.43)	Anxiety (diagnosis)	1.36	(1.07	−1.65)
15	Severe mental illness	2.42	(2.26	−2.58)	Substance misuse	2.20	(2.03	−2.38)	Substance misuse	1.23	(0.95	−1.51)
16	Cholelithiasis	2.02	(1.88	−2.17)	Cholelithiasis	2.11	(1.95	−2.29)	Female infertility	1.18	(0.91	−1.45)
17	Substance misuse	1.98	(1.84	−2.13)	Severe mental illness	2.07	(1.91	−2.24)	Endometriosis	1.05	(0.79	−1.31)
18	Eating disorder	1.88	(1.74	−2.02)	Eating disorder	1.80	(1.65	−1.97)	Hypertension	0.93	(0.69	−1.18)
19	Endometriosis	1.68	(1.55	−1.82)	Inflammatory arthritis	1.40	(1.27	−1.55)	Diabetes mellitus	0.79	(0.57	−1.01)
20	Inflammatory arthritis	1.46	(1.34	−1.59)	Endometriosis	1.31	(1.18	−1.45)	Psoriasis	0.71	(0.50	−0.92)

Common mental health disorders (CMHD): anxiety or depression by prescription (drug phenome using community prescription data) if the woman doesn’t already have a ICD-10 diagnosis code; other chronic headaches: tension headaches, cluster headaches, chronic headaches; other mental health conditions: obsessive compulsive disorder, self-harm, personality disorder, dissociative disorder; other skin conditions: seborrheic dermatitis, rosacea, lichen planus, hidradenitis suppurativa; severe mental illness (SMI): bipolar disorder, schizophrenia, psychosis or meeting drug phenome for SMI; *GORD* gastroesophageal reflux disease, *IBS* Irritable bowel syndrome, *PCOS* polycystic ovarian syndrome

### Combinations of multimorbidity

Table 4 presents the top ten most common combinations of multimorbidity, the most prevalent combinations being *depression and anxiety* in primary care datasets (2.2 and 2.7% of pregnant women in CPRD and SAIL respectively) and *common mental health disorders and asthma* for SMR (3.2%). The presented prevalence is for mutually exclusive multimorbidity combinations, and therefore prevalence for *depression and anxiety* will not include women with depression, anxiety and other health condition/s. When only considering physical conditions, the most common combination was *asthma and allergic rhinoconjunctivitis* (1.7, 2.1 and 2.2% in CPRD, SAIL and SMR respectively).

**Table 4:** Top ten mutually exclusive combinations of multimorbidity in pregnant women

CPRD (UK), ***n*** = 37,641			SAIL (Wales), ***n*** = 27,782			SMR (Scotland), ***n*** = 6099		
**All health conditions**	**n**	**%**	**All health conditions**	**n**	**%**	**All health conditions**	**n**	**%**
Anxiety, Depression	825	2.19%	Anxiety, Depression	748	2.69%	Common mental health disorders (CMHD), Asthma	195	3.20%
Asthma, Allergic rhinoconjunctivitis	370	0.98%	Asthma, Allergic rhinoconjunctivitis	319	1.15%	Peptic ulcer disease/GORD, CMHD	195	3.20%
Depression, Other mental health conditions	214	0.57%	Anxiety, Depression, Other mental health conditions	164	0.59%	Allergic rhinoconjunctivitis, CMHD	145	2.38%
Migraine, Allergic rhinoconjunctivitis	178	0.47%	Allergic rhinoconjunctivitis, Anxiety, Depression	140	0.50%	Allergic rhinoconjunctivitis, Asthma	132	2.16%
Anxiety, Depression, Other mental health conditions	175	0.46%	Asthma, Anxiety, Depression	138	0.50%	Atopic eczema, CMHD	125	2.05%
Asthma, Depression	172	0.46%	Migraine, Anxiety, Depression	128	0.46%	Migraine, CMHD	101	1.66%
Allergic rhinoconjunctivitis, Depression	171	0.45%	Allergic rhinoconjunctivitis, Depression	120	0.43%	Other mental health conditions, CMHD	96	1.57%
Migraine, Depression	161	0.43%	Migraine, Allergic rhinoconjunctivitis	117	0.42%	Asthma, atopic eczema	94	1.54%
Asthma, Anxiety, Depression	140	0.37%	Depression, Other mental health conditions	109	0.39%	Asthma, Peptic ulcer disease/GORD	91	1.49%
Asthma, Migraine^a^	136	0.36%	Asthma, Depression	105	0.38%	Irritable bowel syndrome, CMHD	90	1.48%
**Physical health conditions**	**n**	**%**	**Physical health conditions**	**n**	**%**	**Physical health conditions**	**n**	**%**
Asthma, Allergic rhinoconjunctivitis	626	1.66%	Asthma, Allergic rhinoconjunctivitis	594	2.14%	Asthma, Allergic rhinoconjunctivitis	132	2.16%
Migraine, Allergic rhinoconjunctivitis	318	0.84%	Migraine, Allergic rhinoconjunctivitis	239	0.86%	Asthma, Atopic eczema	94	1.54%
Asthma, Migraine^a^	273	0.73%	Asthma, Migraine	189	0.68%	Asthma, Peptic ulcer disease/GORD	91	1.49%
Allergic rhinoconjunctivitis, Irritable bowel syndrome	175	0.46%	Allergic rhinoconjunctivitis, Irritable bowel syndrome	143	0.51%	Irritable bowel syndrome, Peptic ulcer disease/GORD	79	1.30%
Migraine, Irritable bowel syndrome	175	0.46%	Migraine, Irritable bowel syndrome	136	0.49%	Allergic Rhinitis, Peptic ulcer disease/GORD	76	1.25%
Allergic rhinoconjunctivitis, Other skin conditions	140	0.37%	Asthma, Migraine, Allergic rhinoconjunctivitis	129	0.46%	Atopic eczema, Peptic ulcer disease/GORD	64	1.05%
Asthma, Migraine, Allergic rhinoconjunctivitis	133	0.35%	Migraine, Other chronic headaches	123	0.44%	Allergic rhinoconjunctivitis, Atopic eczema	62	1.02%
Asthma, Irritable bowel syndrome	117	0.31%	Allergic rhinoconjunctivitis, Other chronic headaches	115	0.41%	Migraine, Peptic ulcer disease/GORD	50	0.82%
Migraine, Other skin conditions	98	0.26%	Asthma, Irritable bowel syndrome	110	0.40%	Asthma, Irritable bowel syndrome	45	0.74%
Allergic rhinoconjunctivitis, Other chronic headaches	87	0.23%	Allergic rhinoconjunctivitis, Other skin conditions	98	0.35%	Cholelithiasis, Peptic ulcer disease/GORD	43	0.71%

These multimorbidity combinations are mutually exclusive. For instance, the count for ‘*anxiety and depression’* will include women with exactly these two conditions only, it does not include women with combinations of ‘*anxiety, depression’* and other condition/s

^a^ The percentage of asthma and migraine multimorbidity combination is higher when considering physical health conditions only as it would include combination of these conditions with mental health conditions which are no longer accounted for

*GORD* Gastroesophageal reflux disease

### Prevalence of multimorbidity in pregnant women with selected health conditions

These examples have been provided to illustrate the burden of using the CPRD (UK) pregnancy cohort in 2018. The featured health conditions were the leading causes of maternal deaths in the MBRRACE-UK report [4].

Cardiovascular disease (ischemic heart disease, stroke/transient ischemic attack, heart failure, atrial fibrillation, congenital heart disease, valvular heart disease, cardiomyopathy, hypertension) affected 2.0% (745/37641) of pregnant women, of whom 80.1% (597/745) had multimorbidity. Less than 1 % (246/37641) of pregnant women had a history of venous thromboembolism, among whom 85.8% (211/246) had multimorbidity. Epilepsy affected 1.4% (543/37641) pregnant women, among whom 80.7% (438/543) had multimorbidity.

### Prevalence of selected health conditions by social deprivation and ethnicity

Table 5 presents examples to illustrate the difference in the prevalence of individual health conditions by patient level social deprivation and ethnicity using CPRD (England). Mental health conditions, asthma and epilepsy increased with deprivation. In contrast, some of the common health conditions were more common in the affluent groups, including anxiety, migraine, irritable bowel syndrome, and polycystic ovarian syndrome. For ethnicity, mental health conditions, asthma, migraine, irritable bowel syndrome and psoriasis were more prevalent in White ethnic group; whilst allergic rhinoconjunctivitis and polycystic ovarian syndrome were more prevalent in ethnic minority groups.

**Table 5:** Prevalence of selected health conditions in pregnant women by social deprivation and ethnicity

	**% by patient level IMD quintiles in CPRD (England),** ***n*** **= 13,075**						***P*** **value for** ***X***^**2**^ **test**
**Health conditions**	**1, least deprived**	**2**	**3**	**4**	**5, most deprived**	**Missing**	
	***n*** **= 2326**	***n*** **= 1835**	***n*** **= 1878**	***n*** **= 1853**	***n*** **= 1908**	***n*** **= 3275**	
**Example of health conditions that increased with deprivation**							
***Depression (diagnosis)	20.55	22.29	24.55	24.82	25.58	22.32	< 0.001
*Asthma	12.85	14.22	14.70	15.00	14.83	12.61	0.049
***Other mental health conditions	5.33	6.21	8.31	9.01	10.01	6.93	< 0.001
Psoriasis	3.31	4.31	3.09	4.16	3.41	3.82	0.258
Cardiovascular disease	2.06	2.07	1.70	1.89	2.67	2.29	0.378
***Severe mental illness	1.16	1.58	1.60	2.75	2.99	2.05	< 0.001
**Epilepsy	0.90	1.20	1.65	2.00	2.04	1.04	0.002
Venous thromboembolism	0.77	0.65	0.59	0.49	1.26	0.98	0.073
**Substance misuse	0.52	1.36	1.33	1.46	2.10	1.37	0.001
**Example of health conditions that decreased with deprivation**							
**Anxiety (diagnosis)	20.77	20.05	21.35	18.78	17.82	17.77	0.005
Allergic rhinoconjunctivitis	18.14	16.13	16.03	16.68	15.57	16.15	0.254
*Migraine	14.32	13.35	15.44	12.14	13.16	12.76	0.034
**Irritable bowel syndrome	10.15	8.88	9.16	7.99	6.71	9.04	0.003
**Polycystic ovarian syndrome	8.34	5.78	7.03	6.26	5.29	6.41	0.001
Other skin conditions	6.58	6.70	6.02	6.80	4.98	6.44	0.179
Alcohol misuse	0.82	0.65	0.59	0.81	0.68	0.55	0.820
	**% by ethnicity in CPRD (England)**						***P*** **value for** ***X***^**2**^ **test**
**Health conditions**	**White**	**Black**	**Mixed**	**Others**	**South Asians**	**Missing**	
	***n*** **= 8302**	***n*** **= 490**	***n*** **= 214**	***n*** **= 336**	***n*** **= 843**	***n*** **= 2890**	
**Example of health conditions that were more prevalent in White ethnic group**							
***Depression (diagnosis)	25.20	13.27	20.56	14.29	10.32	23.91	< 0.001
***Anxiety (diagnosis)	21.27	9.80	20.09	10.42	10.08	18.86	< 0.001
***Asthma	14.33	11.22	14.02	6.55	10.79	14.60	< 0.001
***Migraine	14.30	10.20	11.21	8.63	9.85	13.46	< 0.001
***Irritable bowel syndrome	10.07	9.80	6.07	2.68	3.91	7.06	< 0.001
***Other mental health conditions	8.44	5.31	10.28	4.17	1.90	6.92	< 0.001
Other skin conditions	6.75	3.88	5.61	5.06	5.93	5.64	0.050
***Psoriasis	4.05	1.22	3.27	1.49	1.66	3.91	< 0.001
Serious mental illness	2.11	1.63	1.40	0.60	1.19	2.18	0.159
**Substance misuse	1.47	0.82	1.40	0.00	0.00	1.56	0.002
Epilepsy	1.46	0.41	2.80	0.60	1.07	1.52	0.099
Venous thromboembolism	0.93	0.41	1.40	0.60	0.47	0.62	0.309
***Alcohol misuse	0.90	0.00	1.40	0.30	0.00	0.31	< 0.001
**Example of health conditions that were more prevalent in ethnic minority groups**							
***Allergic rhinoconjunctivitis	15.56	22.65	17.29	19.94	16.25	17.65	< 0.001
***Polycystic ovarian syndrome	6.60	5.10	8.88	8.93	9.85	5.33	< 0.001
Cardiovascular disease	2.18	3.88	1.40	1.79	2.14	1.80	0.090

The selected health conditions were the top ten most common conditions in this study or leading causes of maternal death. Other mental health conditions: obsessive compulsive disorder, self-harm, personality disorder, dissociative disorder. Severe mental illness (SMI): bipolar disorder, schizophrenia, psychosis or meeting drug phenome for SMI. Other skin conditions: seborrheic dermatitis, rosacea, lichen planus, hidradenitis suppurativa

*denotes *p* <0.05, ** denotes *p*<0.01, *** denotes *p*<0.001

## Discussion

### Main findings

This study used contemporaneous, routinely collected datasets to study the epidemiology of multimorbidity (defined as having two or more long-term physical or mental health conditions) in pregnant women in the UK. Two in five pregnant women had pre-existing multimorbidity. One in five pregnant women had multimorbidity that were active in the year before pregnancy. Seven in ten pregnant women with multimorbidity had a history of mental health condition/s. In women with conditions that are known to be leading causes of maternal death [4], four in five had pre-existing multimorbidity. Pregnant women with multimorbidity were more likely to be older, multigravid, smoked or have raised BMI preconception.

### Strengths and limitations

This study utilized electronic health records which provided a rich source of data and is generalizable across different settings. It avoided misclassification bias associated with self-reported surveys. However, as with all research that use routine health records, it is subjected to residual confounding and can be limited by the quality and consistency of data entry by clinicians and administrators [14]. We have attempted to improve the accuracy of health conditions ascertainment through the design of our phenome definitions (e.g., using additional age limit and phenomes by prescriptions).

The definition of multimorbidity used in this study was based on simple counting of conditions, without weightings. There is currently no single multimorbidity index that can measure multimorbidity in all settings definitively [15]. The only currently available validated comorbidity index in maternal health research was developed using secondary care data and only included 20 conditions [16], in comparison to the 79 conditions prioritized by our multidisciplinary group and patient representatives. Obesity was analysed as a covariate (BMI categories) in this study; the prevalence of multimorbidity would be higher if obesity was considered a long-term health condition.

#### Utility of the different datasets

Compared with CPRD and SAIL primary care datasets, the prevalence of non-life-threatening health conditions such as allergic rhinoconjunctivitis, migraine, irritable bowel syndrome and, ultimately, multimorbidity was lower in the Scottish secondary care with linked community prescription dataset.

This is likely to reflect that health conditions seen in primary care encompass the whole severity spectrum. Some common conditions, such as anxiety or depression, may only present to primary care, some of which are managed conservatively without prescribed medications. In contrast, the Scottish secondary care and community prescription database would only capture the severe spectrum of a condition that requires hospital attendance or regular prescriptions and may under-estimate the prevalence of multimorbidity. This confirms that primary care health records may be a more comprehensive data source to study pre-existing multimorbidity in pregnant women, and to identify the target at risk population for preconception intervention.

Similar findings were observed in both CPRD, and SAIL add to the validity of these findings. Whilst CPRD offered the benefit of representing data from all four UK nations, SAIL offered a more complete coverage at a population level in Wales with good follow up throughout an individual’s lifetime even when they change GP practices.

Our study highlighted a shortfall in the recording of ethnicity and preconception body mass index, and to a lesser extent, smoking status preconception for pregnant women in routine health records. Patient level data for social deprivation was limited by the availability of data linkage in CPRD. Although sensitivity analysis in the CPRD (England) dataset with imputed ethnicity and IMD showed similar findings with the primary analysis, the interpretation of the association analysis should be taken with caution. In SAIL, pregnancy episodes were detected from the National Community Child Health database (NCCHD), and this does not include pregnancies that resulted in early pregnancy loss; hence the gravidity data generated from this database is likely to be an under-estimation. Historical data from the SMR datasets were available from 2005 to 2019. This meant that if a pregnant woman had a history of a health condition prior to this time period, it may not be captured. This limitation is more likely to affect older women in the SMR pregnancy cohort and may partially account for the lack of association of maternal age with multimorbidity. Further limitations of each dataset are outlined in Additional file 5.

### Results in the context of what is known

#### High prevalence of multimorbidity in pregnant women

The current evidence for the prevalence of multimorbidity in pregnant women is scarce and findings vary widely. This ranged between < 10 to 35% [2, 3, 17, 18]. The high prevalence of multimorbidity in pregnant women in this study is concerning as it is associated with adverse outcomes for mother and child [2–4]. In the latest MBRRACE UK maternal mortality enquiry report, 90% of maternal deaths up to one year post pregnancy occurred in women with multiple health and social problems [4]. MBRRACE has called for national guidance for the management of pregnant women with multiple morbidities and social factors [19]. Recently, the Ockenden report (a high profile UK independent inquiry of maternity services at a local hospital) highlighted the need for involvement of maternal medicine specialist and maternal mental health services for managing women with complex pregnancies [20].

### Clinical implications

Our study provided a current snapshot of how multimorbidity is distributed in the UK in terms of socio-demographics, the health conditions that constitute multimorbidity and the common combinations of health conditions in pregnant women. Mental health conditions were particularly prevalent and contributed to 70% of multimorbidity in pregnant women. Psychiatric causes were amongst the leading cause of maternal death in the UK [4]. Our findings further support the need for integration of mental health services with maternity services and equitable access to perinatal mental health services in the UK [21].

#### Social deprivation, ethnicity and multimorbidity in pregnant women

Post hoc analysis found that some health conditions were more prevalent in affluent pregnant women (e.g., anxiety, irritable bowel syndrome), potentially masking the association of multimorbidity with social deprivation. When these conditions were removed, multimorbidity was associated with social deprivation but this effect was not observed when BMI and smoking status were also adjusted for. This suggests that smoking and obesity may mediate the relationship between social deprivation and multimorbidity in pregnant women.

Many of the topmost common health conditions were more prevalent in White pregnant women, particularly mental health conditions. This may have contributed to the lack of association of ethnic minority groups with multimorbidity. Previous literature reported that people of ethnic minority are less likely to access/receive mental health support/treatment [22, 23]. In addition, stigma associated with mental health conditions was reported to be higher in ethnic minorities [24].

Both observations mean that there could be health care access issues for some of the common health conditions, especially mental health conditions, for people from socially deprived and ethnic minority groups prior to pregnancy. In addition, it strengthens the importance of addressing smoking and obesity preconception especially in pregnant women with multimorbidity from socially deprived groups. Smoking and obesity are two well-known modifiable risk factors for adverse pregnancy outcomes [25, 26], the added impact of multimorbidity is likely to compound this. Further research is required to quantify this, but interventions addressing smoking and obesity may help reduce adverse outcomes in pregnant women with multimorbidity.

### Research implications

Despite the high prevalence of multimorbidity in pregnant women, and the associated adverse outcomes, there is currently a paucity of evidence in this field. The MuM-PreDiCT consortium is a multidisciplinary collaboration across all four nations in the UK, including women with lived experience of multimorbidity and pregnancy. Our next step is to quantify the impact of multimorbidity on pregnancy, maternal and offspring outcomes. This will provide crucial information for women with multimorbidity who are planning a pregnancy and results from the outcome studies may require us to reconsider how we categorize high-risk pregnancy. The ultimate aim is to produce high quality evidence that would guide clinical practice to prevent pregnancy complications and to optimize long-term maternal and offspring health for pregnant women with multimorbidity.

## Conclusion

A significant proportion of women enter pregnancy with pre-existing multimorbidity, especially with mental health condition/s. Amongst pregnant women with health conditions known to be leading causes of maternal death, prevalence of multimorbidity was high. Pregnant women with multimorbidity were more likely to smoke and have a raised BMI and support maybe required to address this. There may be health care access inequalities for some health conditions, especially mental health conditions in pregnant women from deprived or ethnic minority groups.

## Supplementary Information

**Additional file 1.** Cohort selection and data quality checks.

**Additional file 2.** Read codes and International Classification of Disease-version 10 (ICD-10) codes for health conditions.

**Additional file 3.** Phenome definitions of health conditions.

**Additional file 4.** The RECORD statement.

**Additional file 5.** Limitations of CPRD, SAIL, SMR.

**Additional file 6: Figure 1.** Flow chart for selection of study population.

**Additional file 7: Table 1.** Practice level index of multiple deprivation (IMD) quintile by nations in the CPRD 2018 pregnancy cohort.

**Additional file 8: Table 2.** Percentage of pregnant women by the total morbidity count in CPRD, SAIL, SMR in 2018.

**Additional file 9: Table 3.** Prevalence of pre-existing multimorbidity in pregnant women in CPRD, SAIL, SMR in 2018 by women’s characteristics.

**Additional file 10: Table 4.** Sensitivity analysis of CPRD England study cohort (*n* = 13,075) with imputed ethnicity and deprivation data.

**Additional file 11: Table 5.** Post hoc logistic regression with multimorbidity defined using list of 31 conditions from Barnet et al’s paper.

**Additional file 12: Table 6.** Post hoc logistic regression removing conditions that were associated with less deprived IMD quintiles in CPRD England study cohort (*n* = 13,075).

**Additional file 13: Table 7.** Prevalence of individual health conditions in pregnant women aged 15–49 years in CPRD, SAIL, SMR in 2018.

## Data Availability

The data that support the findings of this study are available from CPRD, SAIL and the HIC at the University of Dundee but restrictions apply to the availability of these data, which were used under license for the current study, and so are not publicly available.

## Abbreviations

BMI: Body mass index
CMHD: Common mental health disorder
CPRD: Clinical Practice Research Datalink
GORD: Gastroesophageal reflux disease
GP: General practitioner
HIC: Health Informatics Centre
IBS: Irritable bowel syndrome
IMD: Index of Multiple Deprivation
NCCHD: National Community Child Health database
NHS: National Health Service
OR: Odds ratio
PCOS: Polycystic ovarian syndrome
PPI: Patient and Public Involvement
Ref: Reference
SAIL: Secure Anonymized Information Linkage
SMI: Severe mental illness
SMR: Scottish Morbidity Records
SOP: Standard Operating Procedures
UK: United Kingdom
