# Supplementary material for: Epidemiology of pre-existing multimorbidity in pregnant women in the UK in 2018: a population-based cross-sectional study
Source: BMC Pregnancy Childbirth. 2022 Feb 11;22:120. doi: 10.1186/s12884-022-04442-3 (PMC8840793; doi:10.1186/s12884-022-04442-3)
Supplement: Supplementary file 1 — Additional file 1. Cohort selection and data quality checks. [file 12884_2022_4442_MOESM1_ESM.docx]

# Additional File 1: Cohort selection and data quality checks

Cohort selection

- Index pregnancy is the pregnancy with a start date from 1^st^ Jan 2018 to 31^st^ December 2018.
- When a woman has more than one pregnancy episode in that time frame, the first recorded pregnancy will be used
- Age at pregnancy start date of 15 to 49 years old
- Women of these pregnancies need to have at least one year worth of data recorded preceding index pregnancy

Pregnancy start date

- England - CPRD Pregnancy Register generated pregnancy start dates using primary care pregnancy records following a hierarchical algorithm (2)
- Wales – obtained from National Community Child Health Dataset (NCCHD), when data is not available from NCCHD, pregnancy start date was estimated as 40 weeks before the offspring’s date of birth
- Scotland – last menstrual period date for index pregnancy

CPRD GOLD Pregnancy Register (UK)

- Acceptable patient metric and Up To Standard (UTS) time as practice metric as defined by CPRD (3)
- Death date should be after index pregnancy start date
- Date the patient transferred out of the data-contributing practice should be after index pregnancy start date
- Date of the last data collection for the data-contributing practice should be after index pregnancy start date
- Patient’s first registration date should be at least one year before index pregnancy start date
- UTS date should be at least one year before index pregnancy start date

SAIL (Wales)

- Mothers and children with valid status codes from anonymized matching. The codes that were utilized were:

- 1 National health service (NHS) number passes check digit test

- 2 NHS number derived through external linkage, i.e., Clinical Research Network match on Patient Episode Database for Wales

- 4 Surname, first name, post code, date of birth and gender code match exactly to the Administrative Register

- 39 Surname, post code, date of birth and gender code match exactly to the Administrative Register. First name matches on Lexicon (known variants) or Fuzzy Matching probability >= 0.9.

- Patients need to have a full year of continuous GP practice/s registration in the year prior to conception. A patient’s GP practice/s registration was considered to be continuous if there was no more than 30 days gap between registration with a new GP practice.
- Codes with event dates prior to the week of the patient’s birth or after index pregnancy date were not considered valid for this study.
- Patient’s death date must not be prior to the index pregnancy date.

Scotland (SMR)

- Anonymized linked dataset within a Safe Haven environment was created and maintained using internationally accepted privacy-preserving protocols by Health Informatics Centre (HIC).
- Death date should be after index pregnancy start date
- Date the patient transferred out of the data-contributing health board should be after index pregnancy start date
- Date of the last data collection for the data-contributing health board should be after index pregnancy start date
- Patient’s registration date in the health board should be at least one year before index pregnancy start date
