# Supplementary material for: Epidemiology of pre-existing multimorbidity in pregnant women in the UK in 2018: a population-based cross-sectional study
Source: BMC Pregnancy Childbirth. 2022 Feb 11;22:120. doi: 10.1186/s12884-022-04442-3 (PMC8840793; doi:10.1186/s12884-022-04442-3)
Supplement: Supplementary file 2 — Additional file 2. Read codes and International Classification of Disease-version 10 (ICD-10) codes for health conditions. [file 12884_2022_4442_MOESM2_ESM.docx]

# Additional File 2: Read codes and International Classification of Disease-version 10 (ICD-10) codes for health conditions

<https://github.com/mumpredict/Read-codes-and-ICD-10-codes>

These diagnostic codes were based on existing literature and code list repositories (4-8), and when not available, were generated by clinicians in the research team
