# Supplementary material for: Epidemiology of pre-existing multimorbidity in pregnant women in the UK in 2018: a population-based cross-sectional study
Source: BMC Pregnancy Childbirth. 2022 Feb 11;22:120. doi: 10.1186/s12884-022-04442-3 (PMC8840793; doi:10.1186/s12884-022-04442-3)
Supplement: Supplementary file 5 — Additional file 5. Limitations of CPRD, SAIL, SMR. [file 12884_2022_4442_MOESM5_ESM.docx]

# Additional File 5: Limitations of CPRD, SAIL, SMR

**CPRD**

Linked patient level deprivation data were only available for 75% of the study cohort in England. Although practice level IMD was available for all four nations with no missing data, nation specific practice level IMD cannot be combined due to differences in the IMD domains; therefore, only English data were presented for the association analysis for CPRD. Parity (number of pregnancies that progressed beyond 24 weeks) were not readily available in the CPRD pregnancy register and gravidity was used instead across all three datasets.

**SAIL**

Pregnancy episodes were detected from the National Community Child Health database (NCCHD) and thus does not include pregnancies that resulted in early pregnancy loss. The gravidity generated from the pregnancy episodes identified from the NCCHD is likely to be an under-estimation. Sensitive data, such as human immunodeficiency virus infection and infertility, were not available in the SAIL databank, but is unlikely to have a large effect on the prevalence of multimorbidity.

**SMR**

Historical data from the SMR datasets used in this study were available from 2005-2019, community prescription data from NHS Fife was available from 2009 onwards. This meant that if a pregnant woman had a history of a health condition prior to this time period, it may not be captured. This, together with the fact that secondary care data are more likely to capture the severe spectrum of health conditions, may have led to the lower prevalence of multimorbidity compared to primary care datasets. This limitation is more likely to affect older women in the SMR pregnancy cohort and may partially account for the lack of association of maternal age with multimorbidity. As the Scottish SMR dataset relied on community prescription data to define certain health conditions, this can lead to misclassification.

**References**

1. Barnett K, Mercer SW, Norbury M, Watt G, Wyke S, Guthrie B. Epidemiology of multimorbidity and implications for health care, research, and medical education: a cross-sectional study. Lancet (London, England). 2012;380(9836):37-43.

2. Minassian C, Williams R, Meeraus WH, Smeeth L, Campbell OMR, Thomas SL. Methods to generate and validate a Pregnancy Register in the UK Clinical Practice Research Datalink primary care database. Pharmacoepidemiology and drug safety. 2019;28(7):923-33.

3. Herrett E, Gallagher AM, Bhaskaran K, Forbes H, Mathur R, van Staa T, et al. Data Resource Profile: Clinical Practice Research Datalink (CPRD). International Journal of Epidemiology. 2015;44(3):827-36.

4. Charlson ME, Pompei P, Ales KL, MacKenzie CR. A new method of classifying prognostic comorbidity in longitudinal studies: development and validation. Journal of chronic diseases. 1987;40(5):373-83.

5. Elixhauser A, Steiner C, Harris DR, Coffey RM. Comorbidity Measures for Use with Administrative Data. Medical Care. 1998;36(1):8-27.

6. Primary Care Unit University of Cambridge. CPRD at Cambridge - code lists [cited 2020 10 December]. Available from: <https://www.phpc.cam.ac.uk/pcu/research/research-groups/crmh/cprd_cam/codelists/v11/>.

7. University of Manchester. ClinicalCodes.org: An online clinical codes repository to improve validity and reproducibility of medical database research [cited 2020 10 December]. Available from: <https://clinicalcodes.rss.mhs.man.ac.uk/>.

8. Kuan V, Denaxas S, Gonzalez-Izquierdo A, Direk K, Bhatti O, Husain S, et al. A chronological map of 308 physical and mental health conditions from 4 million individuals in the English National Health Service. The Lancet Digital health. 2019;1(2):e63-e77.

9. Abel KM, Hope H, Swift E, Parisi R, Ashcroft DM, Kosidou K, et al. Prevalence of maternal mental illness among children and adolescents in the UK between 2005 and 2017: a national retrospective cohort analysis. The Lancet Public health. 2019;4(6):e291-e300.

10. Mental Health Foundation. Mental health statistics: the most common mental health problems [cited 2020 29th December]. Available from: <https://www.mentalhealth.org.uk/statistics/mental-health-statistics-most-common-mental-health-problems>.

11. Dunkel Schetter C, Tanner L. Anxiety, depression and stress in pregnancy: implications for mothers, children, research, and practice. Curr Opin Psychiatry. 2012;25(2):141-8.

12. Clinical Knowledge Summary. Depression [cited 2020 29th December]. Available from: <https://cks.nice.org.uk/topics/depression/management/new-or-initial-management/>.
