# Supplementary material for: Epidemiology of pre-existing multimorbidity in pregnant women in the UK in 2018: a population-based cross-sectional study
Source: BMC Pregnancy Childbirth. 2022 Feb 11;22:120. doi: 10.1186/s12884-022-04442-3 (PMC8840793; doi:10.1186/s12884-022-04442-3)

Additional Figure 1: Flow chart for selection of study population

CPRD (UK)

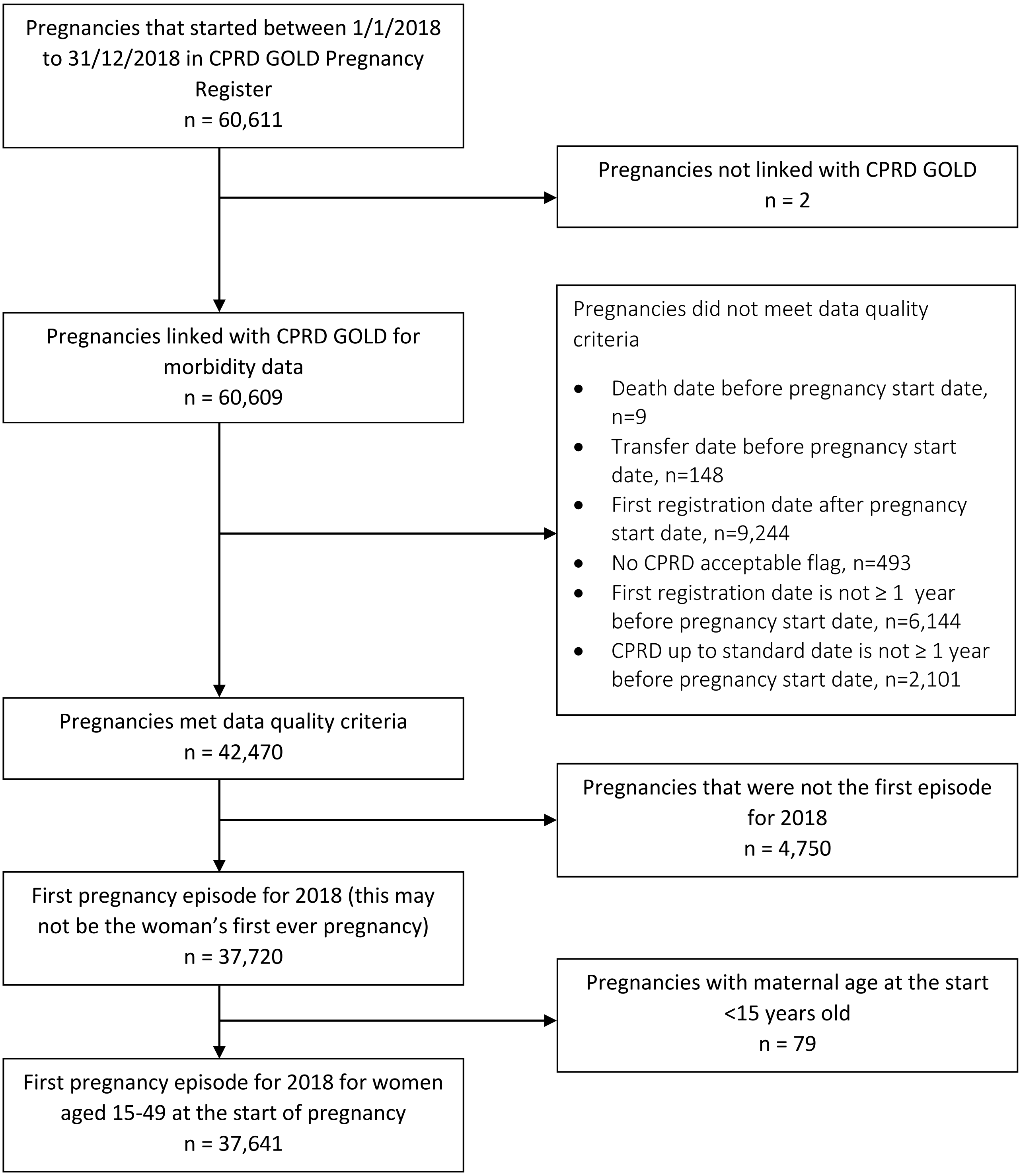

SAIL (Wales)

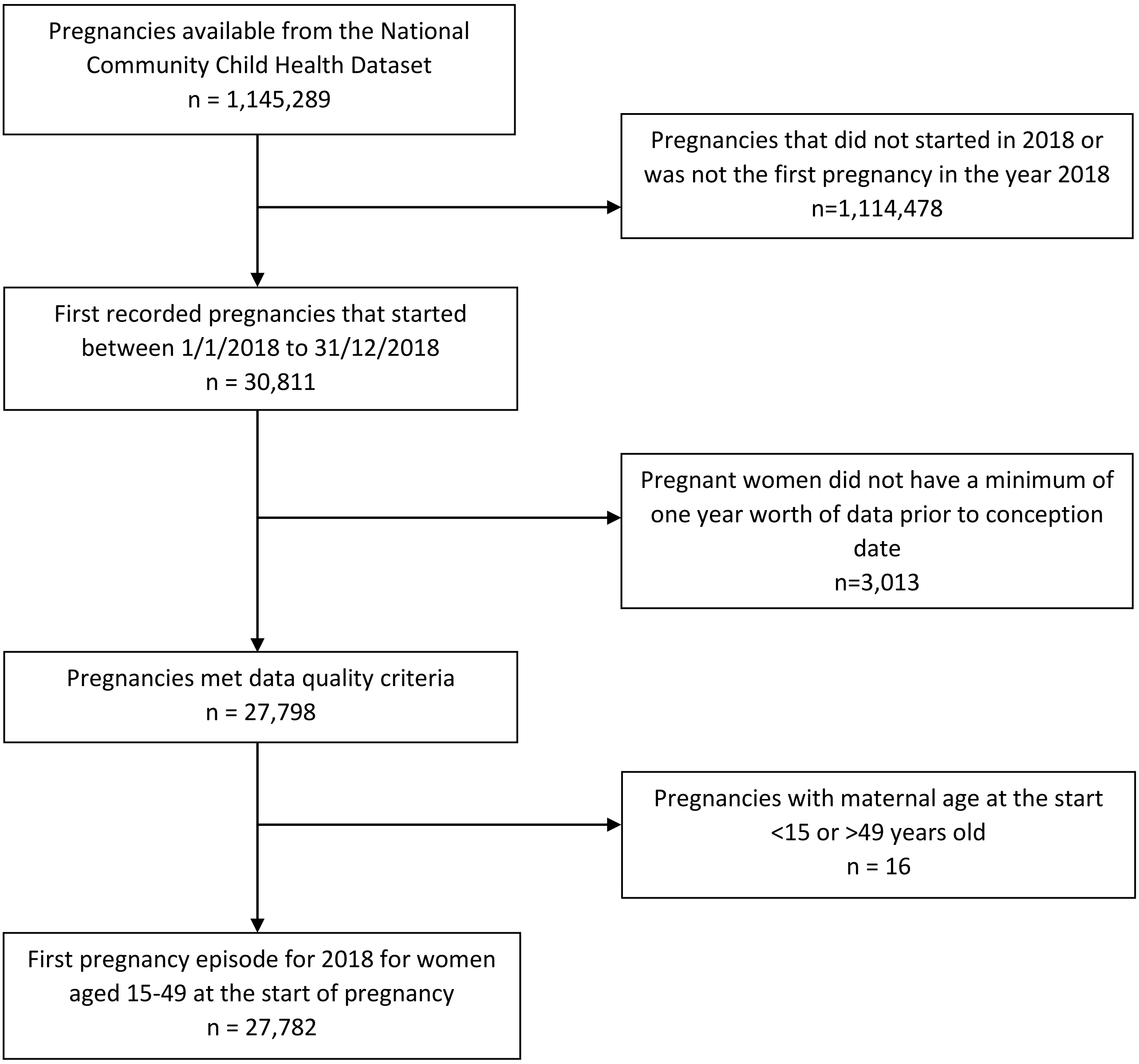
Pregnancies available from the National Community Child Health Dataset n = 1,145,289
Pregnancies that did not started in 2018 or was not the first pregnancy in the year 2018 n=1,114,478
First recorded pregnancies that started between 1/1/2018 to 31/12/2018 n = 30,811
Pregnant women did not have a minimum of one year worth of data prior to conception date n=3,013
Pregnancies met data quality criteria n = 27,798
Pregnancies with maternal age at the start <15 or >49 years old n = 16
First pregnancy episode for 2018 for women aged 15-49 at the start of pregnancy n = 27,782

SMR (Scotland: Tayside and Fife)

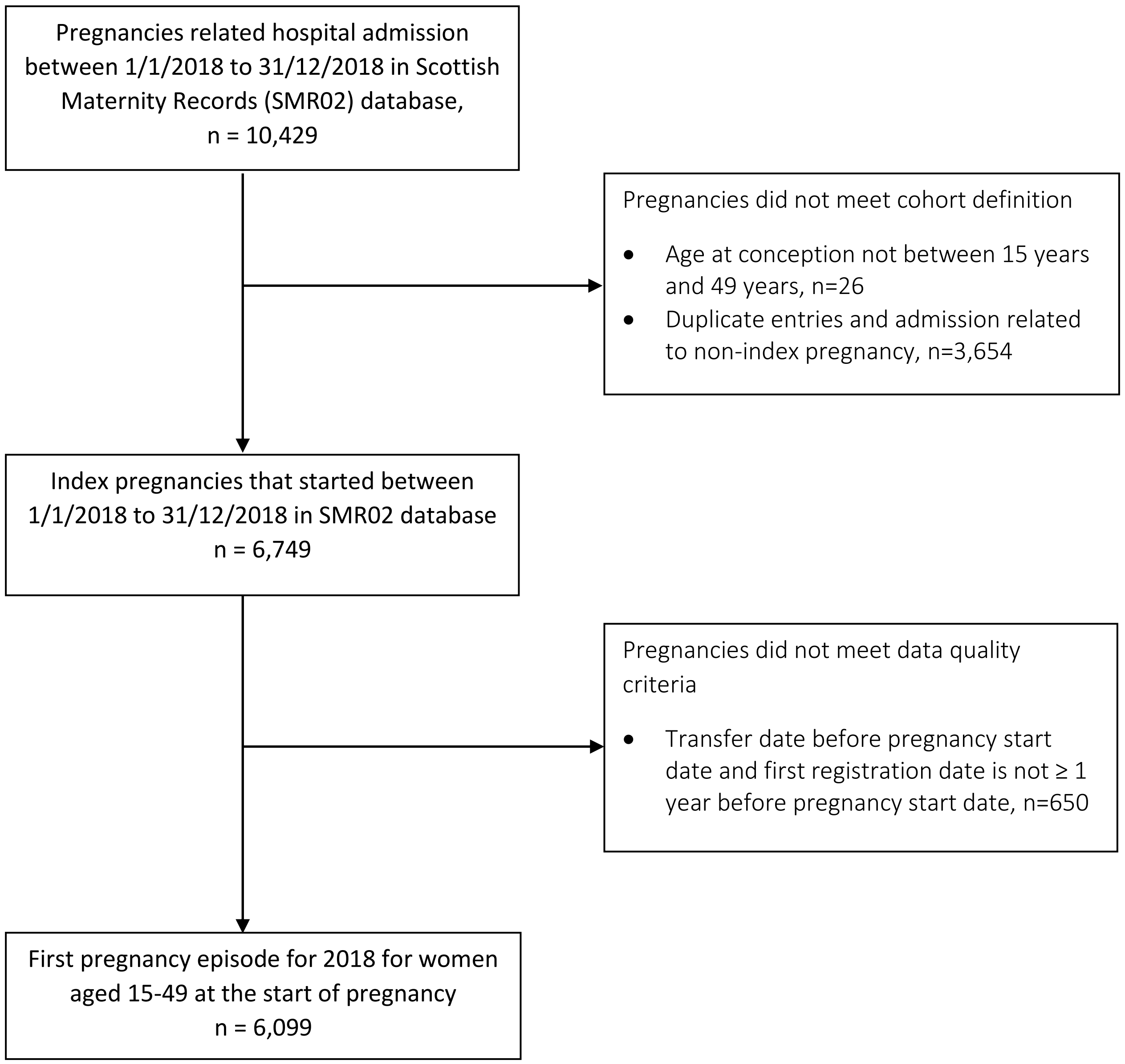

Supplement: Supplementary file 6 — Additional file 6: Figure 1. Flow chart for selection of study population. [file 12884_2022_4442_MOESM6_ESM.pdf]
