# Supplementary material for: Epidemiology of pre-existing multimorbidity in pregnant women in the UK in 2018: a population-based cross-sectional study
Source: BMC Pregnancy Childbirth. 2022 Feb 11;22:120. doi: 10.1186/s12884-022-04442-3 (PMC8840793; doi:10.1186/s12884-022-04442-3)
Supplement: Supplementary file 7 — Additional file 7: Table 1. Practice level index of multiple deprivation (IMD) quintile by nations in the CPRD 2018 pregnancy cohort. [file 12884_2022_4442_MOESM7_ESM.pdf]

Additional Table 1: Practice level index of multiple deprivation (IMD) quintile by nations in the CPRD 2018 pregnancy cohort

	Patient level*, n (%)		Practice level, n (%)							
**Index of multiple deprivation quintile**	**England (n=13075)**		**England (n=13075)**		**Northern Ireland (n=2984)**		**Scotland (n=12559)**		**Wales (n=9023)**	
1, least deprived	2326	17.79%	2274	17.39%	284	9.52%	2142	17.06%	1472	16.31%
2	1835	14.03%	2247	17.19%	392	13.14%	2842	22.63%	801	8.88%
3	1878	14.36%	2288	17.50%	718	24.06%	2214	17.63%	2003	22.20%
4	1853	14.17%	2462	18.83%	277	9.28%	2690	21.42%	2244	24.87%
5, most deprived	1908	14.59%	3804	29.09%	1313	44.00%	2671	21.27%	2503	27.74%
Missing	3275	25.05%	-	-	-	-	-	-	-	-

*Available for England only
