# Supplementary material for: Epidemiology of pre-existing multimorbidity in pregnant women in the UK in 2018: a population-based cross-sectional study
Source: BMC Pregnancy Childbirth. 2022 Feb 11;22:120. doi: 10.1186/s12884-022-04442-3 (PMC8840793; doi:10.1186/s12884-022-04442-3)
Supplement: Supplementary file 8 — Additional file 8: Table 2. Percentage of pregnant women by the total morbidity count in CPRD, SAIL, SMR in 2018. [file 12884_2022_4442_MOESM8_ESM.docx]

# Additional Table 2. Percentage of pregnant women by the total morbidity count in CPRD, SAIL, SMR in 2018

| **Number of pre-existing long-term health conditions per pregnant woman** | **Percentage of pregnant women, % (95% confidence intervals)** | | |
| --- | --- | --- | --- |
|  | **CPRD, n=37641** | **SAIL, n=27782** | **SMR, n=6099** |
| 0 | 29.70 (29.24 - 30.17) | 31.94 (31.39 - 32.49) | 61.99(60.66 - 63.10) |
| 1 | 26.09 (25.64 - 26.53) | 21.90 (21.41 - 22.39) | 18.33 (17.36 - 19.30) |
| 2 | 19.03 (18.63 - 19.43) | 17.71 (17.26 - 18.16) | 10.18 (9.42 - 10.94 |
| 3 | 11.71 (11.38 - 12.04) | 12.54 (12.16 - 12.94) | 5.08 (4.53 - 5.63) |
| 4 | 6.56 (6.31 - 6.81) | 7.47 (7.16 - 7.78) | 2.43 (2.04 - 2.82) |
| 5 | 3.51 (3.33 - 3.70) | 4.16 (3.93 - 4.41) | 1.02 (0.77 - 1.27) |
| 6 | 1.91 (1.77 - 2.05) | 2.26 (2.09 - 2.45) | 0.59 (0.40 - 0.78) |
| 7 | 0.85 (0.76 - 0.95) | 1.08 (0.96 - 1.20) | 0.20 (0.09 - 0.31) |
| ≥8 | 0.65 (0.57 - 0.74) | 0.95 (0.84 - 1.07) | 0.29 (0.16 - 0.42) |
