# Supplementary material for: Epidemiology of pre-existing multimorbidity in pregnant women in the UK in 2018: a population-based cross-sectional study
Source: BMC Pregnancy Childbirth. 2022 Feb 11;22:120. doi: 10.1186/s12884-022-04442-3 (PMC8840793; doi:10.1186/s12884-022-04442-3)
Supplement: Supplementary file 9 — Additional file 9: Table 3. Prevalence of pre-existing multimorbidity in pregnant women in CPRD, SAIL, SMR in 2018 by women’s characteristics. [file 12884_2022_4442_MOESM9_ESM.pdf]

Additional Table 3. Prevalence of pre-existing multimorbidity in pregnant women in CPRD, SAIL, SMR in 2018 by women's characteristics

Characteristics	Percentage of pregnant women affected by multimorbidity, % (95% confidence intervals)		
	CPRD (UK), n=37641	SAIL (Wales), n=27782	SMR (Scotland), n=6099
**All pregnant women**	44.21 (43.71 - 44.71)	46.17 (45.58 - 46.75)	19.79 (18.79 - 20.81)
**Age categories (5 yearly)**			
15-19	27.39 (25.66 - 29.17)	30.38 (28.09 -32.75)	16.11 (12.74 - 19.98)
20-24	40.81 (39.62 - 42.01)	41.72 (40.39 -43.05)	22.23 (19.86 - 24.75)
25-29	44.84 (43.87 - 45.81)	45.69 (44.63 -46.75)	20.77 (18.93 - 22.70)
30-34	45.11 (44.16 - 46.06)	48.83 (47.74 -49.93)	18.56 (16.76 - 20.46)
35-39	49.85 (48.57 - 51.13)	52.61 (50.95 -54.26)	19.18 (16.51 - 22.07)
40-44	53.15 (50.52 - 55.77)	58.21 (54.16 -62.18)	16.67 (10.87 - 23.95)
45-49	58.99 (51.38 - 66.29)	62.86 (44.92 -78.53)	23.07 (5.03 - 53.81)
**Gravidity**			
1	36.81 (35.93 - 37.70)	42.33 (41.48 -43.18)	16.00 (14.34 - 17.78)
2	41.02 (40.05 - 42.00)	46.79 (45.81 -47.78)	17.22 (15.58 - 18.95)
3	46.24 (45.05 - 47.44)	52.52 (50.79 -54.25)	19.82 (17.51 - 22.29)
4	49.00 (47.44 - 50.56)	58.16 (55.09 -61.19)	25.17 (21.69 - 28.91)
≥5	59.33 (58.03 - 60.63)	66.73 (62.49 -70.78)	33.89 (30.25 - 37.88)
Missing	- - -	- - -	25.00 (0.63 - 80.59)
**Ethnicity**			
Asian / South Asian[a]	33.78 (31.17 - 36.47)	33.25 (28.75 -38.00)	10.74 (6.30 - 16.85)
Black	46.97 (43.79 - 50.16)	24.16 (18.07 -31.13)	17.39 (4.95 - 38.78)
Mixed	40.00 (34.46 - 45.74)	44.63 (35.59 -53.94)	25.00 (3.18 - 65.09)
Other	33.52 (29.50 - 37.73)	24.45 (19.03 -30.55)	14.29 (7.83 - 23.19)
White	45.97 (45.30 - 46.65)	50.92 (50.17 -51.66)	21.22 (20.04 - 22.40)
Missing	42.80 (41.97 - 43.64)	38.90 (37.91 -39.89)	14.16 (11.97 - 16.58)

Characteristics	Percentage of pregnant women affected by multimorbidity, % (95% confidence intervals) CPRD, n=37641	SAIL, n=27782	SMR, n=6099
**BMI (kg/m^2)**			
Underweight (<18.5)	45.85 (43.02 - 48.70)	50.82 (48.05 -53.58)	22.83 (14.72 - 32.75)
Normal Weight (18.5-24.9)	42.80 (41.99 - 43.61)	49.33 (48.32 -50.34)	15.70 (13.88 - 17.65)
Overweight (25-29.9)	47.80 (46.71 - 48.90)	53.57 (52.26 -54.88)	19.60 (17.20 - 22.18)
Obese (30+)	55.57 (54.41 - 56.73)	60.52 (59.20 -61.83)	26.97 (24.56 - 29.50)
Missing	30.52 (29.42 - 31.63)	20.25 (19.24 -21.29)	18.35 (16.77 - 20.01)
**Smoking**			
Non-Smoker	39.92 (39.28 - 40.57)	41.25 (40.29 -42.21)	15.85 (14.64 - 17.13)
Ex-Smoker	52.13 (50.82 - 53.43)	56.07 (54.98 -57.16)	21.67 (18.96 - 24.57)
Smoker	54.55 (53.46 - 55.63)	60.56 (59.37 -61.74)	32.37 (29.53 - 35.31)
Missing	17.82 (15.78 - 20.01)	4.57 (3.85 -5.38)	17.97 (15.43 - 20.72)
**Patient level deprivation quintiles (IMD)**	**Patient level IMD data only available for England[b]**		
1, least deprived	45.79 (43.75 - 47.84)	49.30 (48.07 -50.52)	11.36 (9.14 - 13.90)
2	44.47 (42.18 - 46.78)	45.70 (44.37 -47.03)	14.53 (12.44 - 16.83)
3	45.69 (43.42 - 47.97)	46.64 (45.22 -48.07)	19.20 (16.78 - 21.81)
4	45.33 (43.05 - 47.63)	43.01 (41.47 -44.55)	25.54 (23.14 - 28.05)
5, most deprived	44.81 (42.56 - 47.08)	46.22 (44.63 -47.81)	23.96 (21.70 - 26.33)
Missing	43.27 (41.56 - 44.98)	43.89 (42.17 -45.62)	18.90 (16.18 - 21.83)

[a] South Asian for CPRD, Asian for SAIL and SMR

[b] Aggregate IMD quintiles cannot be provided for UK as each nation has its specific IMD; data presented here is patient level IMD for England only (n=13075).

BMI: body mass index, CPRD: Clinical Practice Research Datalink, IMD: Index of Multiple Deprivation, SAIL: The Secure Anonymized Information Linkage databank, SMR: Scottish Morbidity Records
