# Supplementary material for: Epidemiology of pre-existing multimorbidity in pregnant women in the UK in 2018: a population-based cross-sectional study
Source: BMC Pregnancy Childbirth. 2022 Feb 11;22:120. doi: 10.1186/s12884-022-04442-3 (PMC8840793; doi:10.1186/s12884-022-04442-3)
Supplement: Supplementary file 10 — Additional file 10: Table 4. Sensitivity analysis of CPRD England study cohort (n = 13,075) with imputed ethnicity and deprivation data. [file 12884_2022_4442_MOESM10_ESM.pdf]

Additional Table 4. Sensitivity analysis of CPRD England study cohort (n=13,075) with imputed ethnicity and deprivation data

Imputed data

Missing ethnicity data in CPRD Gold was imputed with ethnicity data from linked Hospital Episodes Statistics. Missing patient level Index of Multiple Deprivation (IMD) was imputed with practice level IMD from the same patient.

Additional Table 4a. Imputed data for ethnicity and deprivation data in CPRD England study data

Characteristics	Original data		Imputed data	
	n	%	n	%
**Ethnicity**				
Black	490	3.75	525	4.02
Mixed	214	1.64	252	1.93
Other	336	2.57	420	3.21
South Asians	843	6.45	912	6.98
White	8302	63.50	10126	77.45
Missing	2890	22.10	840	6.42
**Patient level deprivation quintiles (IMD)**				
1, least deprived	2326	17.79	2905	22.22
2	1835	14.03	2275	17.40
3	1878	14.36	2279	17.43
4	1853	14.17	2816	21.54
5, most deprived	1908	14.59	2800	21.41
Missing	3275	25.05	-	-

Additional Table 4b. Logistic regression of multimorbidity with imputed ethnicity and IMD data in CPRD England cohort

Characteristics	CPRD (England), n=13075					
	Unadjusted OR (95% CI)			Adjusted OR (95% CI)		
**Age categories (5 yearly)**						
15-19	Ref	-	-	Ref	-	-
20-24	1.60	(1.34	1.90)	1.19	(0.99	1.44)
25-29	1.80	(1.52	2.12)	1.23	(1.02	1.48)
30-34	1.84	(1.56	2.17)	1.28	(1.07	1.54)
35-39	1.95	(1.64	2.32)	1.30	(1.07	1.58)
40-44	2.55	(2.04	3.20)	1.65	(1.29	2.10)
45-49	2.98	(1.74	5.11)	1.82	(1.04	3.18)
**Gravidity**						
1	Ref	-	-	Ref	-	-
2	1.07	(0.97	1.18)	0.98	(0.89	1.08)
3	1.35	(1.22	1.50)	1.18	(1.05	1.31)
4	1.52	(1.35	1.72)	1.29	(1.14	1.47)
≥5	2.11	(1.90	2.35)	1.68	(1.50	1.89)
**Ethnicity**						
Black	0.72	(0.60	0.86)	0.69	(0.57	0.83)
Mixed	0.88	(0.69	1.14)	0.94	(0.73	1.22)
Other	0.49	(0.40	0.61)	0.54	(0.44	0.67)
South Asian	0.60	(0.52	0.69)	0.65	(0.56	0.75)
White	Ref	-	-	Ref	-	-
Missing	0.54	(0.46	0.62)	0.62	(0.53	0.73)
**BMI (kg/m^2)**						
Underweight (<18.5)	0.89	(0.73	1.07)	0.93	(0.76	1.13)
Normal Weight (18.5-24.9)	Ref	-	-	Ref	-	-
Overweight (25-29.9)	1.20	(1.10	1.31)	1.16	(1.06	1.27)
Obese (30+)	1.69	(1.53	1.86)	1.60	(1.44	1.76)
Missing	0.60	(0.54	0.67)	0.74	(0.65	0.83)
**Smoking**						
Non-Smoker	Ref	-	-	Ref	-	-
Ex-Smoker	1.62	(1.47	1.78)	1.40	(1.27	1.55)
Smoker	1.69	(1.54	1.84)	1.57	(1.43	1.73)
Missing	0.31	(0.24	0.41)	0.50	(0.37	0.66)
**Patient level deprivation quintiles (IMD)**						
1, least deprived	Ref	-	-	Ref	-	-
2	0.88	(0.79	0.99)	0.88	(0.79	0.99)
3	1.03	(0.93	1.15)	0.97	(0.86	1.08)
4	0.94	(0.85	1.05)	0.89	(0.79	0.99)
5, most deprived	0.98	(0.88	1.08)	0.90	(0.81	1.01)
