# Supplementary material for: Epidemiology of pre-existing multimorbidity in pregnant women in the UK in 2018: a population-based cross-sectional study
Source: BMC Pregnancy Childbirth. 2022 Feb 11;22:120. doi: 10.1186/s12884-022-04442-3 (PMC8840793; doi:10.1186/s12884-022-04442-3)
Supplement: Supplementary file 11 — Additional file 11: Table 5. Post hoc logistic regression with multimorbidity defined using list of 31 conditions from Barnet et al’s paper. [file 12884_2022_4442_MOESM11_ESM.docx]

# Additional Table 5. Post hoc logistic regression with multimorbidity defined using list of 31 conditions from Barnet et al’s paper.

Our study did not observe that multimorbidity was associated with increasing levels of social deprivation. To explore whether this is due to the list of conditions we used to define multimorbidity, we repeated the logistic regression in a model where multimorbidity was defined by the list of conditions used in Barnet et al’s seminal paper (1).

Barnet et al’s study found that multimorbidity increased with social deprivation across different age groups. However, their study included both male and female as well as the elderly population. We included the list of conditions that overlapped with our study and where possible used similar phenome definitions.

**Additional Table 5a. List of conditions in the post hoc analysis with multimorbidity defined using list of 31 conditions from Barnet et al’s paper.**

| **31 conditions that were included in this post hoc analysis** | **Conditions that were not included in this post hoc analysis** | |
| --- | --- | --- |
|  | **Present in Barnet et al’s study but not in this study** | **Present in this study but not in Barnet et al’s study** |
| Hypertension | Painful conditions | Congenital heart disease |
| Active depression | Treated constipation | Valvular disease |
| Active asthma | Diverticular disease | Cardiomyopathy |
| Ischemic heart disease/ myocardial infraction | Peripheral vascular disease | Autoimmune skin conditions |
| Peptic ulcer disease | Prostate disorders | Other skin conditions |
| Diabetes mellitus | Glaucoma | Allergic rhinoconjunctivitis |
| Hyper/hypothyroidism | Dementia | Cataract |
| Systemic lupus erythematosus/Inflammatory arthritis/Spondylarthritis | Chronic sinusitis | Diabetic retinopathy |
| Profound deafness | Parkinson’s disease | Inflammatory eye disease |
| Chronic obstructive pulmonary disease |  | Retinal detachment |
| Active anxiety |  | Coeliac disease |
| Irritable bowel syndrome |  | Cholelithiasis |
| Active cancer in last 5 years |  | Polycystic ovarian syndrome |
| Substance misuse |  | Endometriosis |
| Alcohol misuse |  | Leiomyoma (fibroids) |
| Stroke / transient ischemic attack |  | Female infertility |
| Chronic kidney disease |  | Venous thromboembolism |
| Atrial fibrillation |  | Primary thrombocytopenia |
| Heart failure |  | Haemophilia |
| Epilepsy |  | Pernicious anaemia |
| Severe mental illness |  | Sickle cell disease |
| Active eczema / psoriasis |  | Neurodevelopmental disorder (Attention deficit hyperactive disorder, autistic spectrum disorder) |
| Inflammatory bowel disease |  | Other mental health conditions |
| Active migraine |  | Scoliosis |
| Severe blindness |  | Vertebrae disorders |
| Eating disorder |  | Osteoarthritis |
| Learning disability |  | Chronic back pain |
| Bronchiectasis |  | Osteoporosis |
| Multiple sclerosis |  | Other chronic headaches |
| Chronic viral hepatitis |  | Spina bifida |
| Chronic liver disease |  | Idiopathic intracranial hypertension |
|  |  | Peripheral neuropathy |
|  |  | Obstructive sleep apnoea |
|  |  | Interstitial lung disease |
|  |  | Pulmonary hypertension |
|  |  | Cystic fibrosis |
|  |  | Sarcoidosis |
|  |  | Urolithiasis |
|  |  | Hyperparathyroidism |
|  |  | Pituitary disorder |
|  |  | Adrenal benign tumours |
|  |  | Human immunodeficiency virus infection / acquired immunodeficiency syndrome |
|  |  | Turner’s syndrome |
|  |  | Marfan’s syndrome |
|  |  | Solid organ transplant |

* Active disease phenome definition as outlined in Additional File 3.

**Additional Table 5b. Logistic regression models with multimorbidity defined using 31 conditions in Barnet et al’s paper (1), in CPRD England (n=13,075)**

| **Model** | **Index of multiple deprivation (IMD) quintiles** | **Odds ratio (95% confidence intervals)** | **p value** |
| --- | --- | --- | --- |
| **Model 1**  Patient level IMD | 1, Least deprived | Reference | - |
|  | 2 | 1.09 (0.91 to 1.31) | 0.354 |
|  | 3 | 1.24 (1.03 to 1.48) | 0.021 |
|  | 4 | 1.32 (1.11 to 1.58) | 0.002 |
|  | 5, Most deprived | 1.28 (1.07 to 1.53) | 0.006 |
|  | Missing | 1.06 (0.90 to 1.25) | 0.493 |
| **Model 2**  Model 1 + Maternal age | 1, Least deprived | Reference | - |
|  | 2 | 1.11 (0.92 to 1.33) | 0.279 |
|  | 3 | 1.27 (1.06 to 1.52) | 0.010 |
|  | 4 | 1.36 (1.14 to 1.63) | 0.001 |
|  | 5, Most deprived | 1.34 (1.11 to 1.60) | 0.002 |
|  | Missing | 1.08 (0.91 to 1.27) | 0.376 |
| **Model 3**  Model 2 + Ethnicity | 1, Least deprived | Reference | - |
|  | 2 | 1.11 (0.92 to 1.34) | 0.266 |
|  | 3 | 1.28 (1.07 to 1.54) | 0.008 |
|  | 4 | 1.45 (1.21 to 1.74) | <0.001 |
|  | 5, Most deprived | 1.43 (1.20 to 1.72) | <0.001 |
|  | Missing | 1.10 (0.94 to 1.30) | 0.235 |
| **Model 4**  Model 3 + Gravidity | 1, Least deprived | Reference | - |
|  | 2 | 1.10 (0.91 to 1.33) | 0.302 |
|  | 3 | 1.24 (1.03 to 1.48) | 0.024 |
|  | 4 | 1.35 (1.12 to 1.62) | 0.001 |
|  | 5, Most deprived | 1.30 (1.08 to 1.57) | 0.005 |
|  | Missing | 1.06 (0.90 to 1.25) | 0.504 |
| **Model 5**  Model 4 + Body mass index categories | 1, Least deprived | Reference | - |
|  | 2 | 1.08 (0.89 to 1.30) | 0.435 |
|  | 3 | 1.19 (0.99 to 1.43) | 0.068 |
|  | 4 | 1.26 (1.05 to 1.51) | 0.015 |
|  | 5, Most deprived | 1.22 (1.02 to 1.47) | 0.033 |
|  | Missing | 1.02 (0.86 to 1.20) | 0.809 |
| **Model 6**  Model 5 + Smoking status | 1, Least deprived | Reference | - |
|  | 2 | 1.03 (0.85 to 1.25) | 0.753 |
|  | 3 | 1.10 (0.92 to 1.33) | 0.303 |
|  | 4 | 1.13 (0.94 to 1.36) | 0.194 |
|  | 5, Most deprived | 1.05 (0.87 to 1.27) | 0.583 |
|  | Missing | 0.97 (0.82 to 1.14) | 0.702 |
