# Supplementary material for: Epidemiology of pre-existing multimorbidity in pregnant women in the UK in 2018: a population-based cross-sectional study
Source: BMC Pregnancy Childbirth. 2022 Feb 11;22:120. doi: 10.1186/s12884-022-04442-3 (PMC8840793; doi:10.1186/s12884-022-04442-3)
Supplement: Supplementary file 12 — Additional file 12: Table 6. Post hoc logistic regression removing conditions that were associated with less deprived IMD quintiles in CPRD England study cohort (n = 13,075). [file 12884_2022_4442_MOESM12_ESM.docx]

# Additional Table 6. Post hoc logistic regression removing conditions that were associated with less deprived IMD quintiles in CPRD England study cohort (n=13,075)

This post hoc analysis was performed to further test our hypothesis that the lack of association of multimorbidity with social deprivation may be due to the health conditions used to define multimorbidity.

To identify health conditions that were associated with less deprived social economic status, the list of 79 health conditions in this study was each tested with linear regression against patient level index of multiple deprivation (IMD) quintiles (1 being least deprived, 5 being most deprived, missing values not imputed). IMD quintiles were treated as continuous variables in the linear regression to produce a single effect size.

The following eight health conditions were found to be associated with less deprived socio-economic background. The logistic regression was repeated using the remaining 71 health conditions in the CPRD England dataset.

**Additional Table 6a. Linear regression of eight health conditions that were significantly, negatively associated with the Index of Multiple Deprivation**

| **Health conditions** | **Coefficient** | **95% confidence intervals** | | **p value** |
| --- | --- | --- | --- | --- |
| Endometriosis | -0.004 | -0.006 | -0.002 | <0.001 |
| Irritable bowel syndrome | -0.008 | -0.012 | -0.004 | <0.001 |
| Female infertility | -0.006 | -0.009 | -0.003 | <0.001 |
| Polycystic ovarian syndrome | -0.006 | -0.009 | -0.003 | 0.001 |
| Hyper/hypothyroidism | -0.003 | -0.006 | -0.001 | 0.009 |
| Anxiety | -0.007 | -0.012 | -0.002 | 0.012 |
| Vertebrae disorders | -0.002 | -0.003 | -0.000 | 0.014 |
| Inflammatory arthritis | -0.002 | -0.003 | -0.000 | 0.024 |

**Additional Table 6b. Logistic regression with multimorbidity defined by 71 health conditions in the CPRD England dataset.**

| **Model** | **IMD quintiles** | **Odds ratio (95% confidence intervals)** | **p value** |
| --- | --- | --- | --- |
| **Model 1**  Patient level IMD | 1, Least deprived | Reference | - |
|  | 2 | 1.07 (0.94 to 1.22) | 0.326 |
|  | 3 | 1.12 (0.99 to 1.28) | 0.082 |
|  | 4 | 1.22 (1.07 to 1.39) | 0.002 |
|  | 5, Most deprived | 1.25 (1.10 to 1.42) | 0.001 |
|  | Missing | 1.01 (0.90 to 1.14) | 0.807 |
| **Model 2**  Model 1 + Maternal age | 1, Least deprived | Reference | - |
|  | 2 | 1.08 (0.95 to 1.24) | 0.234 |
|  | 3 | 1.15 (1.01 to 1.31) | 0.041 |
|  | 4 | 1.26 (1.10 to 1.44) | 0.001 |
|  | 5, Most deprived | 1.30 (1.14 to 1.49) | <0.001 |
|  | Missing | 1.03 (0.92 to 1.16) | 0.616 |
| **Model 3**  Model 2 + Ethnicity | 1, Least deprived | Reference | - |
|  | 2 | 1.09 (0.95 to 1.24) | 0.225 |
|  | 3 | 1.16 (1.01 to 1.32) | 0.032 |
|  | 4 | 1.32 (1.15 to 1.50) | <0.001 |
|  | 5, Most deprived | 1.37 (1.20 to 1.57) | <0.001 |
|  | Missing | 1.05 (0.93 to 1.18) | 0.419 |
| **Model 4**  Model 3 + Gravidity | 1, Least deprived | Reference | - |
|  | 2 | 1.08 (0.94 to 1.23) | 0.283 |
|  | 3 | 1.12 (0.98 to 1.28) | 0.103 |
|  | 4 | 1.23 (1.08 to 1.41) | 0.002 |
|  | 5, Most deprived | 1.26 (1.10 to 1.44) | 0.001 |
|  | Missing | 1.01 (0.90 to 1.14) | 0.859 |
| **Model 5**  Model 4 + Body mass index categories | 1, Least deprived | Reference | - |
|  | 2 | 1.05 (0.92 to 1.20) | 0.501 |
|  | 3 | 1.06 (0.93 to 1.21) | 0.392 |
|  | 4 | 1.14 (1.00 to 1.31) | 0.053 |
|  | 5, Most deprived | 1.18 (1.03 to 1.36) | 0.015 |
|  | Missing | 0.97 (0.86 to 1.09) | 0.595 |
| **Model 6**  Model 5 + Smoking status | 1, Least deprived | Reference | - |
|  | 2 | 1.02 (0.89 to 1.17) | 0.773 |
|  | 3 | 1.01 (0.88 to 1.16) | 0.873 |
|  | 4 | 1.07 (0.93 to 1.23) | 0.328 |
|  | 5, Most deprived | 1.08 (0.94 to 1.24) | 0.284 |
|  | Missing | 0.94 (0.83 to 1.06) | 0.308 |
