# Supplementary material for: Epidemiology of pre-existing multimorbidity in pregnant women in the UK in 2018: a population-based cross-sectional study
Source: BMC Pregnancy Childbirth. 2022 Feb 11;22:120. doi: 10.1186/s12884-022-04442-3 (PMC8840793; doi:10.1186/s12884-022-04442-3)
Supplement: Supplementary file 13 — Additional file 13: Table 7. Prevalence of individual health conditions in pregnant women aged 15–49 years in CPRD, SAIL, SMR in 2018. [file 12884_2022_4442_MOESM13_ESM.docx]

# Additional Table 7. Prevalence of individual health conditions in pregnant women aged 15-49 years in CPRD, SAIL, SMR in 2018

|  |  | **Percentages, %** | | |
| --- | --- | --- | --- | --- |
| **No** | **Health conditions^a^** | **CPRD,**  **n=37641** | **SAIL,**  **n=27782** | **SMR,**  **n=6099** |
|  | **Cancer** |  |  |  |
| 1 | All cancers | 0.51 | 0.60 | 0.57 |
|  | *Primary breast cancer* | 0.07 | 0.06 | <0.08 |
|  | *Primary lung cancer* | <0.01 | 0.00 | <0.08 |
|  | *Primary bowel cancer* | <0.01 | <0.02 | <0.08 |
|  | *Primary cervical cancer* | 0.03 | 0.03 | 0.08 |
|  | *Primary ovarian cancer* | <0.01 | 0.03 | <0.08 |
|  | *Primary uterine cancer* | <0.01 | <0.02 | <0.08 |
|  | *Primary thyroid cancer* | 0.05 | 0.03 | <0.08 |
|  | *Primary skin cancer*  *(excluding basal cell carcinoma)* | 0.18 | 0.27 | 0.15 |
|  | *Lymphoma* | 0.07 | 0.08 | <0.08 |
|  | *Leukaemia* | 0.03 | 0.04 | <0.08 |
|  | *Metastatic cancer* | <0.01 | <0.02 | 0.10 |
|  |  |  |  |  |
|  | **Cardiovascular** |  |  |  |
| 2 | Hypertension | 0.87 | 0.67 | 0.93 |
| 3 | Ischemic heart disease | 0.07 | 0.18 | <0.08 |
| 4 | Heart failure | 0.07 | 0.07 | <0.08 |
| 5 | Stroke / transient ischemic attack | 0.20 | 0.17 | <0.08 |
| 6 | Atrial fibrillation | 0.05 | 0.03 | <0.08 |
| 7 | Congenital heart disease | 0.67 | 0.76 | <0.08 |
| 8 | Valvular heart disease | 0.15 | 0.14 | <0.08 |
| 9 | Cardiomyopathy | 0.05 | 0.02 | <0.08 |
|  |  |  |  |  |
|  | **Dermatology** |  |  |  |
| 10 | Atopic eczema | 3.06 | 3.97 | 6.35 |
|  | Atopic eczema (active) | 1.58 | 1.80 | 3.36 |
| 11 | Psoriasis | 3.90 | 3.62 | 0.71 |
|  | Psoriasis (active) | 1.46 | 1.20 | 0.36 |
| 12 | Autoimmune skin conditions | 0.73 | 0.86 | <0.08 |
|  | *Vitiligo* | 0.26 | 0.30 | <0.08 |
|  | *Alopecia areata* | 0.48 | 0.57 | <0.08 |
| 13 | Other skin conditions | 5.47 | 5.71 | 0.31 |
|  | Other skin conditions (active) | 1.46 | 1.43 | - |
|  | *Seborrheic dermatitis* | 2.60 | 3.02 | <0.08 |
|  | *Seborrheic dermatitis (active)* | 0.79 | 0.87 | - |
|  | *Rosacea* | 2.22 | 1.98 | <0.08 |
|  | *Rosacea (active)* | 0.45 | 0.33 | - |
|  | *Hidradenitis suppurativa* | 0.80 | 0.90 | 0.15 |
|  | *Hidradenitis suppurativa (active)* | 0.24 | 0.26 | - |
|  | *Lichen planus* | 0.10 | 0.08 | <0.08 |
|  | *Lichen planus (active)* | 0.01 | <0.02 | - |
|  |  |  |  |  |
|  | **Ear nose throat** |  |  |  |
| 14 | Allergic Rhinoconjunctivitis | 16.35 | 18.53 | 7.56 |
|  | Allergic Rhinoconjunctivitis (active) | 1.27 | 3.17 | 3.34 |
| 15 | Profound deafness | 0.19 | 0.32 | 0.16 |
|  |  |  |  |  |
|  | **Eye** |  |  |  |
| 16 | Severe blindness | 0.02 | 0.21 | <0.08 |
| 17 | Cataract | 0.11 | 0.18 | <0.08 |
| 18 | Diabetic eye disease  *(retinopathy, maculopathy)* | 0.39 | 0.34 | <0.08 |
| 19 | Inflammatory eye conditions  *(uveitis, scleritis, episcleritis)* | 0.55 | 0.65 | <0.08 |
| 20 | Retinal detachment | 0.09 | 0.09 | <0.08 |
|  |  |  |  |  |
|  | **Gastrointestinal** |  |  |  |
| 21 | Inflammatory bowel disease | 0.60 | 0.58 | 0.38 |
|  | *Crohn’s disease* | 0.33 | 0.28 | 0.25 |
|  | *Ulcerative colitis* | 0.31 | 0.34 | 0.18 |
| 22 | Irritable bowel syndrome | 7.97 | 7.83 | 3.16 |
| 23 | Coeliac disease | 0.41 | 0.40 | <0.08 |
| 24 | Peptic ulcer disease  *(or gastroesophageal reflux disease for SMR)* | 0.21 | 0.14 | 6.98 |
| 25 | Cholelithiasis | 2.02 | 2.11 | 2.15 |
| 26 | Chronic liver disease | 0.42 | 0.34 | <0.08^b^ |
|  | *Chronic hepatitis B, C* | 0.22 | 0.13 | <0.08 |
|  | *Autoimmune liver disease* | <0.01 | 0.03 | <0.08 |
|  | *Chronic alcoholic liver disease* | <0.01 | <0.02 | <0.08 |
|  | *Non-alcoholic fatty liver disease* | 0.12 | 0.10 | 0.13^b^ |
|  | *Cirrhosis* | 0.02 | 0.04 | <0.08 |
|  |  |  |  |  |
|  | **Gynaecology** |  |  |  |
| 27 | Polycystic ovarian syndrome | 4.66 | 3.96 | 0.25 |
| 28 | Endometriosis | 1.68 | 1.31 | 1.05 |
| 29 | Leiomyoma | 0.61 | 0.33 | 0.26 |
| 30 | Female infertility | 3.81 | - | 1.18 |
|  |  |  |  |  |
|  | **Haematology** |  |  |  |
| 31 | Venous thromboembolism | 0.65 | 0.60 | 0.10 |
| 32 | Primary thrombocytopenia | 0.14 | 0.19 | <0.08 |
| 33 | Haemophilia | <0.01 | <0.02 | <0.08 |
| 34 | Pernicious anaemia | 0.20 | 0.20 | <0.08 |
| 35 | Sickle cell disease | 0.01 | <0.02 | <0.08 |
|  |  |  |  |  |
|  |  |  |  |  |
|  | **Mental health conditions** |  |  |  |
|  |  |  |  |  |
|  | **Common mental health disorders (diagnosis code)** |  |  |  |
| 36 | Depression | 23.43 | 24.07 | 1.71 |
| 37 | Anxiety | 18.98 | 23.05 | 1.36 |
|  | *Anxiety* | 18.59 | 22.74 | - |
|  | *Post-traumatic stress disorder* | 0.76 | 0.78 | - |
|  | Depression OR Anxiety | 30.97 | 32.26 | - |
|  |  |  |  |  |
|  | **Common mental health disorders (drug phenome)** |  |  |  |
|  | 4 prescriptions in 12 months | 20.28 | 21.99 | 22.66 |
|  | 2 prescriptions, minimum 1 month apart, in 6 months^c^ | 25.23 | 26.33 | 28.43 |
|  |  |  |  |  |
|  | **Common mental health disorders (diagnosis / drug)** |  |  |  |
|  | Diagnosis code OR 4 prescriptions in 12 months for depression or anxiety | 35.69 | 36.22 | - |
|  |  |  |  |  |
|  | **Common mental health disorders (active)** |  |  |  |
|  | CMHD (drug phenome) | - | - | 21.79 |
|  | Depression (diagnosis code + drug) | 11.50 | 12.03 | - |
|  | Anxiety (diagnosis code + drug) | 9.47 | 11.34 | - |
|  |  |  |  |  |
|  | **Severe mental illness (diagnosis code)** |  |  |  |
|  | SMI | 0.71 | 0.75 | 0.46 |
|  | *Bipolar disorder / affective psychosis* | 0.47 | 0.45 | 0.23 |
|  | *Schizophrenia / non-affective psychosis* | 0.33 | 0.40 | 0.28 |
|  |  |  |  |  |
|  | **Severe mental illness (drug phenome)** |  |  |  |
|  | 4 prescriptions in 12 months | 2.12 | 1.81 | 2.26 |
|  | 2 prescriptions, minimum 1 month apart, in 6 months^c^ | 2.23 | 1.87 | 2.28 |
|  |  |  |  |  |
|  | **Severe mental illness (diagnosis/drug)** |  |  |  |
| 38 | Diagnosis code OR 4 prescriptions in 12 months | 2.42 | 2.07 | - |
|  |  |  |  |  |
|  | Improving access to psychological therapies (IAPT, diagnosis code) | 0.32 | 0.00 | - |
|  |  |  |  |  |
| 39 | Eating disorder | 1.88 | 1.80 | <0.08 |
|  |  |  |  |  |
|  | **Alcohol misuse/dependency** |  |  |  |
|  | Diagnosis code | 0.95 | 2.25 | 0.59 |
| 40 | Diagnosis code OR 4 prescriptions in 12 months | 0.97 | 2.25 | 0.62 |
|  | Diagnosis code OR 2 prescriptions, minimum 1 month apart, in 6 months)^c^ | 0.98 | 2.26 | - |
|  |  |  |  |  |
|  | **Substance misuse/dependency** |  |  |  |
|  | Diagnosis code | 1.98 | 2.20 | 0.89 |
| 41 | Diagnosis code OR 4 prescriptions in 12 months | 1.98 | 2.20 | 1.23 |
|  | Diagnosis code OR 2 prescriptions, minimum 1 month apart, in 6 months^c^ | 1.98 | 2.20 | - |
|  |  |  |  |  |
| 42 | Neurodevelopmental disorder | 0.79 | 0.93 | 0.38 |
|  | *Attention deficit hyperactivity disorder* | 0.39 | 0.57 | 0.10 |
|  | *Autism* | 0.17 | 0.13 | <0.08 |
|  | *Learning difficulty* | 0.29 | 0.32 | 0.15 |
|  |  |  |  |  |
| 43 | Other mental health conditions | 8.85 | 9.43 | 4.84 |
|  | Other mental health conditions (active) | 1.78 | 1.83 | - |
|  | *Obsessive compulsive disorder* | 0.73 | 0.70 | <0.08 |
|  | *Obsessive compulsive disorder (active)* | 0.07 | 0.07 | - |
|  | *Personality disorder* | 1.09 | 0.98 | 0.36 |
|  | *Self-harm/suicide* | 7.83 | 8.40 | 4.64 |
|  | *Self-harm/suicide (active)* | 0.63 | 0.60 | - |
|  | *Dissociative disorder* | 0.10 | 0.27 | <0.08 |
|  |  |  |  |  |
|  | **Rheumatology** |  |  |  |
| 44 | Systemic lupus erythematosus | 0.09 | 0.07 | <0.08 |
| 45 | Spondylarthritis | 0.19 | 0.16 | <0.08 |
|  | *Psoriatic arthritis* | 0.14 | 0.11 | <0.08 |
|  | *Ankylosing spondylitis* | 0.05 | 0.05 | <0.08 |
| 46 | Inflammatory arthritis | 1.46 | 1.40 | 0.57 |
|  | *Rheumatoid arthritis* | 0.16 | 0.12 | 0.16 |
|  | *Raynaud’s disease* | 1.25 | 1.23 | 0.08 |
|  | *Sjogren’s disease* | 0.03 | 0.02 | <0.08 |
|  | *Systemic sclerosis* | <0.01 | <0.02 | <0.08 |
|  | *Primary systemic vasculitis* | 0.03 | 0.04 | <0.08 |
| 47 | Ehlers’s Danlos Syndrome (EDS) Type 3 (Hypermobile EDS) | 0.53 | 0.45 | 0.13 |
|  |  |  |  |  |
|  | **Orthopaedic** |  |  |  |
| 48 | Scoliosis | 0.60 | 0.54 | 0.13 |
| 49 | Vertebrae disorder | 0.78 | 0.84 | 0.23 |
|  | *Intervertebral disc disorder* | 0.57 | 0.54 | 0.18 |
|  | *Spondylolisthesis* | 0.05 | 0.06 | <0.08 |
|  | *Spondylosis* | 0.17 | 0.23 | <0.08 |
|  | *Collapsed vertebrae* | 0.02 | 0.03 | <0.08 |
|  | *Spinal stenosis* | <0.01 | 0.03 | <0.08 |
| 50 | Chronic back pain | 0.74 | 0.67 | 0.67 |
| 51 | Osteoporosis | 0.09 | 0.13 | <0.08 |
| 52 | Osteoarthritis | 0.31 | 0.29 | 0.28 |
|  |  |  |  |  |
|  | **Neurological** |  |  |  |
| 53 | Migraine | 12.71 | 13.47 | 3.69 |
|  | Migraine (active) | 3.08 | 1.62 | 2.49 |
| 54 | Other chronic headaches | 3.53 | 6.60 | 0.13 |
|  | Other chronic headaches (active) | 0.29 | 0.52 | - |
|  | *Tension type headache* | 3.22 | 6.35 | 0.11 |
|  | *Tension type headache (active)* | 0.25 | 0.49 | - |
|  | *Cluster headache* | 0.29 | 0.23 | <0.08 |
|  | *Cluster headache (active)* | 0.04 | 0.03 | - |
|  | *Other chronic headaches* | 0.08 | 0.17 | 0.13 |
|  | *Other chronic headaches (active)* | 0.02 | <0.02 | - |
| 55 | Epilepsy | 1.44 | 1.30 | 1.57 |
| 56 | Multiple sclerosis | 0.15 | 0.09 | <0.08 |
| 57 | Spina bifida | 0.08 | 0.11 | - |
| 58 | Idiopathic intracranial hypertension | 0.19 | 0.22 | 0.11 |
| 59 | Peripheral neuropathy | 0.46 | 0.62 | 0.16 |
| 60 | Somatoform disorder | 1.14 | 1.14 | 0.21 |
|  | *Chronic fatigue syndrome* | 0.36 | 0.48 | <0.08 |
|  | *Fibromyalgia* | 0.61 | 0.53 | 0.13 |
|  | *Chronic Pain* | 0.25 | 0.41 | <0.08 |
|  |  |  |  |  |
|  | **Respiratory** |  |  |  |
| 61 | Asthma | 14.63 | 17.17 | 10.49 |
|  | Asthma (active) | 7.09 | 7.13 | 8.26 |
| 62 | Chronic obstructive pulmonary disease | 0.06 | 0.08 | 0.23 |
| 63 | Obstructive sleep apnoea | 0.28 | 0.28 | <0.08 |
| 64 | Interstitial lung disease / pulmonary fibrosis | <0.01 | <0.02 | <0.08 |
| 65 | Pulmonary hypertension | 0.02 | <0.02 | <0.08 |
| 66 | Bronchiectasis | 0.10 | 0.21 | <0.08 |
| 67 | Cystic fibrosis | 0.02 | 0.02 | <0.08 |
| 68 | Sarcoidosis | 0.05 | 0.05 | <0.08 |
|  |  |  |  |  |
|  | **Renal** |  |  |  |
| 69 | Chronic kidney disease stage (CKD) 3-5 | 0.12 | 0.09 | <0.08 |
|  | *CKD by diagnosis codes* | 0.10 | 0.06 | <0.08 |
|  | *CKD by eGFR* | 0.06 | 0.06 | - |
|  | *Dialysis* | 0.03 | <0.02 | <0.08 |
| 70 | Urolithiasis | 0.40 | 0.33 | 0.46 |
|  |  |  |  |  |
|  | **Endocrine** |  |  |  |
| 71 | Diabetes mellitus (DM) | 0.99 | 0.84 | 0.79 |
|  | *Type 1 DM* | 0.56 | 0.49 | - |
|  | *Type 2 DM* | 0.71 | 0.68 | - |
| 72 | Thyroid disorder | 3.34 | 2.45 | 3.12 |
|  | *Hyperthyroidism* | 0.73 | 0.55 | - |
|  | *Hypothyroidism* | 2.82 | 2.07 | - |
| 73 | Pituitary disorder | 0.35 | 0.27 | <0.08 |
|  | *Prolactinoma* | 0.07 | 0.06 | - |
| 74 | Adrenal benign tumour | <0.01 | 0.02 | <0.08 |
| 75 | Hyperparathyroidism | 0.02 | <0.02 | <0.08 |
|  |  |  |  |  |
|  | **Other** |  |  |  |
| 76 | Turner syndrome | 0.02 | 0.02 | <0.08 |
| 77 | Marfan syndrome | 0.03 | <0.02 | <0.08 |
| 78 | Solid organ transplant | 0.03 | 0.09 | <0.08 |
| 79 | Human immunodeficiency virus infection (HIV) / acquired immunodeficiency syndrome (AIDS) | 0.06 | - | <0.08 |

**^a^** Constituent health conditions may not add up to the total in the combined categories either because there may be overlaps or not all constituent health conditions have been presented in this table.

^b^ For SMR, chronic liver disease includes chronic hepatitis B & C, alcoholic liver disease, autoimmune liver disease, cirrhosis

^c^ Sensitivity analysis of the prevalence of individual health conditions when using a drug phenome of 2 prescriptions, minimum 1 month apart, in 6 months

NB: active diseases were active in the last 12 months
